# Epigenetic changes induced by in utero dietary challenge result in phenotypic variability in successive generations of mice

**DOI:** 10.1038/s41467-022-30022-2

**Published:** 2022-05-05

**Authors:** Mathew Van de Pette, Andrew Dimond, António M. Galvão, Steven J. Millership, Wilson To, Chiara Prodani, Gráinne McNamara, Ludovica Bruno, Alessandro Sardini, Zoe Webster, James McGinty, Paul M. W. French, Anthony G. Uren, Juan Castillo-Fernandez, William Watkinson, Anne C. Ferguson-Smith, Matthias Merkenschlager, Rosalind M. John, Gavin Kelsey, Amanda G. Fisher

**Affiliations:** 1 Lymphocyte Development & Epigenetic Memory Groups, MRC London Institute of Medical Sciences, Imperial College London, Hammersmith Hospital Campus, Du Cane Road, London, W12 0NN UK; 2 Epigenetics Programme, The Babraham Institute, Cambridge, CB22 3AT UK; 3 Institute of Animal Reproduction and Food Research of PAS, Department of Reproductive Immunology and Pathology, Olsztyn, Poland; 4 Centre for Trophoblast Research, University of Cambridge, Cambridge, CB2 3EG UK; 5 Department of Medicine, Imperial College London, Hammersmith Hospital Campus, Du Cane Road, London, W12 0NN UK; 6 Whole Animal Physiology and Imaging, MRC London Institute of Medical Sciences, Imperial College London, Hammersmith Hospital Campus, Du Cane Road, London, W12 0NN UK; 7 Transgenics and Embryonic Stem Cell Laboratory, MRC London Institute of Medical Sciences, Imperial College London, Hammersmith Hospital Campus, Du Cane Road, London, W12 0NN UK; 8 Photonics Group, Department of Physics, Imperial College London, South Kensington Campus, London, SW7 2AZ UK; 9 Cancer Genomics Group, MRC London Institute of Medical Sciences, Imperial College London, Hammersmith Hospital Campus, Du Cane Road, London, W12 0NN UK; 10 Department of Genetics, University of Cambridge, Downing Street, Cambridge, CB2 3EH UK; 11 Cardiff School of Biosciences, Cardiff University, Cardiff, CF10 3AX UK; 12 Wellcome-MRC Institute of Metabolic Science-Metabolic Research Laboratories, Cambridge, CB2 0QQ UK

**Keywords:** Epigenetic memory, Oogenesis, DNA methylation, Imprinting, Bioluminescence imaging

## Abstract

Transmission of epigenetic information between generations occurs in nematodes, flies and plants, mediated by specialised small RNA pathways, modified histones and DNA methylation. Similar processes in mammals can also affect phenotype through intergenerational or trans-generational mechanisms. Here we generate a luciferase knock-in reporter mouse for the imprinted *Dlk1* locus to visualise and track epigenetic fidelity across generations. Exposure to high-fat diet in pregnancy provokes sustained re-expression of the normally silent maternal *Dlk1* in offspring (loss of imprinting) and increased DNA methylation at the somatic differentially methylated region (*sDMR*). In the next generation heterogeneous *Dlk1* mis-expression is seen exclusively among animals born to F1-exposed females. Oocytes from these females show altered gene and microRNA expression without changes in DNA methylation, and correct imprinting is restored in subsequent generations. Our results illustrate how diet impacts the foetal epigenome, disturbing canonical and non-canonical imprinting mechanisms to modulate the properties of successive generations of offspring.

## Abstract (web summary)

Here the authors show that a high-fat diet in pregnant mice can release silencing of the imprinted Dlk1 locus in multiple generations of offspring. They found that this occurs via changes in microRNA expression at the locus of interest, as well as transcriptional changes across the genome, in the developing oocytes.

## Introduction

Epigenetic gene regulation, and the processes that enable information that is not strictly encoded by DNA to be transmitted to offspring, is the subject of intense study in model organisms^1–8^ and in man^9,10^. Genomic imprinting is an epigenetically regulated process that restricts mammalian gene expression in a parent-of-origin-specific manner^11,12^. Mono-allelic gene expression is initiated by differential DNA methylation of parental germlines but is often reinforced postfertilisation by the acquisition of additional epigenetic features that help sustain appropriate allelic expression (or silencing) within somatic tissues^13–15^. As a group, imprinted genes are critical for controlling embryonic growth and placental development^13,14,16^ and have key roles later in postnatal life, where they influence metabolism, neurogenesis and behaviour^17–19^. The expression of imprinted genes is tightly regulated and subtle changes in expression often lead to profound changes in phenotype^17,20,21^.

*Dlk1* is a prototypic paternally expressed, imprinted gene that is broadly expressed in the mid-gestation embryo but becomes increasingly restricted in the adult to subpopulations of cells in the adrenal and pituitary glands, skeletal muscle, liver and brain^22–26^. Paternally restricted expression of *Dlk1* is associated with reciprocal expression of maternal *Gtl2* (*Meg3*), *Rian* (*Meg8*), anti-sense *Rtl1* (*Rtl1as*), as well as clusters of intergenic microRNAs (*Mirg*) that collectively comprise one of the largest microRNA (miR) clusters in the genome^27^. Molecular studies have shown that imprinting of the *Dlk1-Dio3* region is primarily regulated by a differentially methylated region (DMR), the *IG-DMR*, that shows selective methylation on the paternally inherited allele. Localised methylation across the *Dlk1* somatic DMR (*sDMR*) and *Gtl2 sDMR* occurs after fertilization and reinforces allelic marking to ensure expression of *Dlk1* and *Gtl2* from paternal and maternal alleles respectively^24,28,29^.

Luciferase-based imaging offers a powerful non-invasive approach to visualise gene expression longitudinally in mammals^30,31^. We have previously shown that targeting luciferase into endogenous imprinted genes, such as *Cdkn1c*, enables allelic expression to be monitored in living mice throughout their lifespan. These reporter mice have also been used to show that in utero exposure to chromatin-modifying drugs, or dietary stress, can induce a sustained loss of imprinting (LOI) in offspring^30^.

Here we ask whether diet-induced deregulation of imprinting can be inherited across generations. Using a bespoke mouse luciferase reporter for allelic *Dlk1* expression, we show that F1 animals that were exposed to a maternal high-fat diet (HFD) in utero experience a sustained loss of *Dlk1* imprinting. F2 offspring born to exposed F1 mothers exhibit deregulated and ectopic expression of *Dlk1*. We show that this intergenerational change in phenotype stems from alterations in the transcriptional profile of oocytes from embryos that were exposed to HFD in utero.

## Results

### *Dlk1-FlucLacZ* reporter mice show imprinted *Dlk1* expression

**Fig. 1:**
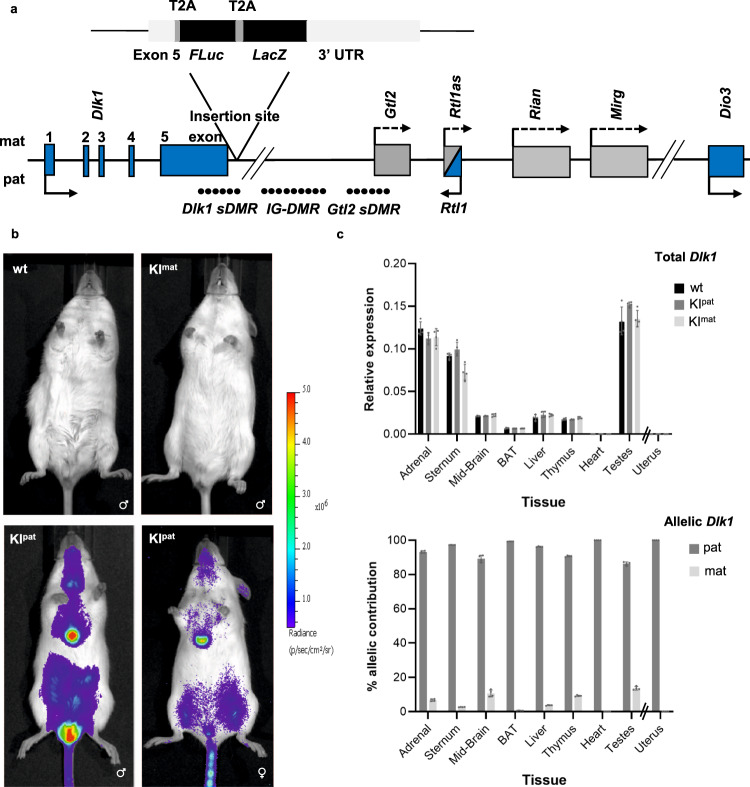
Generation and characterisation of reporter mice for imprinted *Dlk1* expression. **a** Schematic of the mouse *Dlk1-Dio3* imprinted locus showing reporter insertion. Three differentially methylated regions (DMRs) that regulate imprinted expression of the cluster are indicated (closed circles represent methylated CpGs, *IG-DMR*, *Dlk1 sDMR* and *Gtl2 sDMR*) and the position of maternally expressed (light grey) and paternally expressed (blue) genes are shown. Arrows depict transcriptional direction, with solid lines representing protein-coding genes and striped lines representing non-coding transcripts. In the *Dlk1-FLucLacZ* reporter line, *firefly Luciferase* (*FLuc*) and *β–galactosidase* (*LacZ*) were knocked into the endogenous *Dlk1* locus, with T2A sites, downstream of exon 5. **b** Bioluminescence (BL, blue) was detected in 8-week-old (P56) male (lower panel, left) and female mice (lower panel, right) after paternal transmission of the reporter (KI^pat^). Strong BL signal was evident in the thymus, central sternum and testes. Minimal signal was detected in animals after maternal reporter transmission (KI^mat^, upper panel, right) or in wild-type animals (wt, upper panel, left). **c** *Dlk1* expression analysed by QRT-PCR (upper panel) was compared in different tissues from P56 male mice that inherited the reporter paternally (KI^pat^, dark grey), maternally (KI^mat^, light grey), or in non-transgenic controls (wt, black). Uterus samples from age-matched female mice were also analysed. Expression levels were normalized to *β-Actin*, *18S* and *Hprt* expression (bars show the geometric mean of relative expression, error bars represent the geometric standard deviation (geometric SD)). Genotype had no significant effect on *Dlk1* expression (Two-way ANOVA on delta-Ct values (Tissue *p* < 0.0001, Genotype *p* = 0.86, Interaction *p* = 0.98); *N* = 4 + 4 + 4 individual mice). Allelic *Dlk1* analysis in KI^mat^ mice (lower panel), using primers that distinguish the reporter from the wt allele, showed a strong bias for paternal allele expression (dark grey) compared to maternal allele expression (light grey). (Bars indicate the mean contribution from each allele ±SD; *N* = 4 + 4 individual mice). Source data are provided as a Source Data file.

We generated a mouse reporter in which *firefly luciferase* (*FLuc*) and *β-galactosidase* (*LacZ*) were knocked into the 3’UTR of the endogenous *Dlk1* gene. The targeting strategy (Fig. 1a and methods) employed T2A sites to generate *Dlk1-FLucLacZ* RNA species under the control of the *Dlk1* promoter, that upon translation and self-cleavage at T2A sites, produce individual luciferase, β-galactosidase and Dlk1 proteins. To confirm that luciferase activity accurately reports *Dlk1* expression in these mice, we performed a series of bioluminescence (BL) imaging, immunohistology and molecular analyses. The engineered *Dlk1* reporter allele showed faithful paternal expression^17,26^ with BL signal exclusively detected in *Dlk1-FLucLacZ* adult mice that inherited the reporter paternally (KI^pat^, Fig. 1b) and evident in the brain, abdomen, testes, liver, adrenal glands and central sternum. No BL signal was seen in *Dlk1-FLucLacZ* mice inheriting the reporter maternally (KI^mat^), or in wild-type (wt) control animals. Tissue-specific and allelic *Dlk1* expression in the *Dlk1-FLucLacZ* reporter mice was verified by QRT-PCR. As shown in Fig. 1c (upper panel), *Dlk1* transcript levels were similar in reporter and non-transgenic animals, consistent with minimal locus disruption. As anticipated, transgene-derived *Dlk1* expression was detected in adrenal, midbrain, liver, testes, and in brown adipose tissue (BAT), but not in heart or uterine tissue. A strong bias in expression from the paternal allele was confirmed in each of these tissues (Fig. 1c, lower panel) consistent with the imprinted expression of *Dlk1* that has been reported previously^22,32–34^. To further validate these molecular analyses, we examined Dlk1 immunolabelling and LacZ staining on adult tissue from control (wt) and reporter (*Dlk1-FLucLacZ*) mice. As anticipated, Dlk1 is readily detected in pituitary and adrenal tissues, but not in kidney or heart (Fig. S1a). Similarly, in KI^pat^ reporter mice Dlk1 labelling was evident in the pituitary, adrenal, and liver tissue (Fig. S1b, left column) and undetected in heart. Examination of sequential sections of KI^pat^ tissues stained for LacZ showed a close correspondence between LacZ stain and Dlk1 distribution. No LacZ staining was evident in equivalent tissues derived from adult KI^mat^ or wt mice (Fig. S1b, right-hand columns). These data highlight the fidelity of the *Dlk1-FLucLacZ* mouse line to report tissue-specific and imprinted *Dlk1* expression.

To verify that DNA methylation was correctly maintained in these reporter mice we performed bisulphite analysis across the *Dlk1 sDMR*, *IG-DMR* and *Gtl2 sDMR*, comparing *Dlk1-FLucLacZ* KI^pat^ and wt samples of adult liver. Methylation levels were indistinguishable at each of the *Dlk1* DMR regions analysed (Fig. S1c). An independent method was also used to verify these results. As shown in Fig. S1d pyrosequencing analysis at the *Dlk1 sDMR* confirmed similar levels of DNA methylation in *Dlk1-FLucLacZ* KI^pat^, *Dlk1-FLucLacZ* KI^mat^ and wt samples. Taken together these results support the view that *Dlk1-FLucLacZ* accurately reports endogenous *Dlk1* expression and that reporter insertion at the *Dlk1-Dio3* locus had not substantially altered the methylation of regulatory DMRs.

**Fig. 2:**
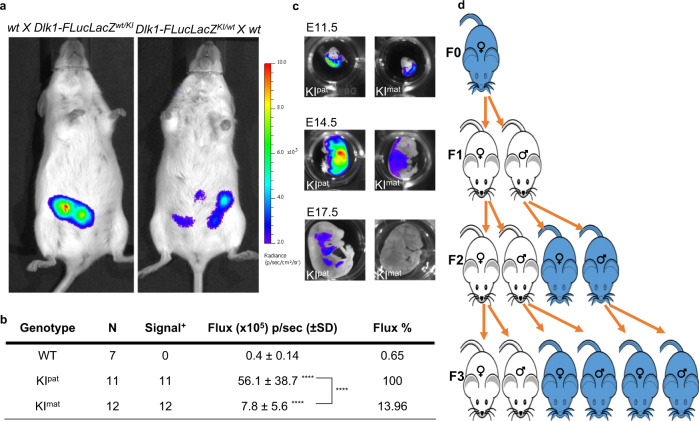
Inheritance of imprinted *Dlk1* reporter expression in embryos and across generations. **a** BL signal (blue) detected in *Dlk1-FLucLacZ* pregnancies arising from KI^pat^ (left) and KI^mat^ (right) transmission showed greater surface signal in KI^pat^ pregnancies (E11.5). **b** Quantification of BL signal (Flux) detected in E11.5 *Dlk1-FLucLacZ* embryos following dissection, demonstrating higher levels of signal from KI^pat^ than KI^mat^ embryos. BL signal in wt and KI^mat^ embryos is shown as a percentage of the average KI^pat^ signal (number of embryos (*N*) indicated in table; One-way ANOVA on log-transformed data (*p* < 0.0001); results of Holm-Šídák’s multiple comparisons follow-up test are shown for comparisons to wt mice, and between KI^pat^/KI^mat^ mice as indicated: ****adjusted *p* (*p*adj) < 0.0001). Source data are provided as a Source Data file. **c** BL imaging of embryos at different stages (E11.5, E14.5 and E17.5) showed progressive reduction in signal (blue) in both KI^pat^ (left panel) and KI^mat^ (right panel) through gestation; signal was readily detected in E11.5 and E14.5 KI^pat^ and KI^mat^ embryos, but at later stages (E17.5) was only seen after paternal transmission. Signal intensity scales are equalised between images. **d** Transmission of mono-allelic imprinted *Dlk1* reporter expression in four generations (F0, F1, F2, F3); upon paternal inheritance of *Dlk1-FLucLacZ* the reporter was expressed (blue), while maternal inheritance resulted in reporter silencing (white). Imprinting was predictably re-set across generations, through both germlines (a minimum of two independent litters were analysed per generation and reciprocal cross).

BL imaging studies performed during pregnancy revealed abdominal reporter signal in pregnant mice carrying embryos with a paternally inherited *Dlk1*-reporter (Fig. 2a, left) and dissection and ex vivo imaging confirmed expression in E11.5 embryos (Figs. 2b, c and S2a). Transiently, from E11.5 to E14.5 a weaker signal was seen in embryos with a maternally inherited *Dlk1*-reporter, but this was extinguished by E17.5 (Fig. 2c). Temporal reinforcement of mono-allelic *Dlk1* expression during development is consistent with prior reports^32,35,36^. To examine tissue-specific reporter expression in developing embryos, whole-mount staining for LacZ and optical projection tomography was performed on E11.5 embryos (Fig. S2b, Movies S1–3). LacZ staining showed abundant *Dlk1-FLucLacZ* reporter expression in KI^pat^ embryos with signal prominent in the forebrain, cartilage, developing lung, liver, pancreas and tongue (Fig. S2b), consistent with the developmental expression of Dlk1 that has been previously reported^22,37^. Within the KI^pat^ forebrain, immunolabelling confirmed that luciferase expression (red) was restricted to a subset of cells co-labelled with anti-Dlk1 antibody (green, Fig. S2c).

To verify that imprint erasure and re-setting occurs correctly in the *Dlk1-FLucLacZ* reporter line, we tracked BL activity in animals established from reciprocal crosses spanning four generations (*N* > 6 per reciprocal cross and generation). Figure 2d illustrates these results, showing that epigenetic inheritance in the *Dlk1-FLucLacZ* colony followed the expected pattern for a paternally expressed imprinted gene. Collectively these results reaffirm that *Dlk1-FLucLacZ* mice are reliable models of imprinted *Dlk1* expression.

### HFD in pregnancy causes loss of *Dlk1* imprinting in offspring

**Fig. 3:**
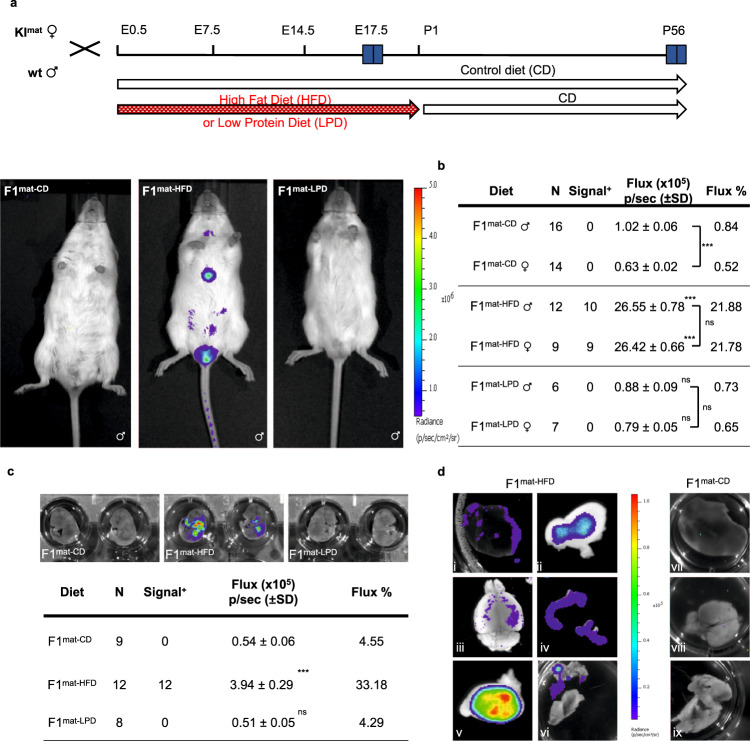
Exposure to high fat diet in utero results in loss of *Dlk1* imprinting in offspring. **a** Temporal scheme of experimental breeding, dietary regime and bioluminescent image analysis. Offspring inheriting *Dlk1-FLucLacZ* maternally (KI^mat^) were generated by mating wt males with heterozygous *Dlk1-FLucLacZ* females; upon detection of a vaginal plug pregnant females were maintained on a control (CD) diet or switched to low protein diet (LPD), or high fat diet (HFD), for the duration of the pregnancy. At birth, all animals were maintained on CD and BL imaging was performed on reporter offspring at the times indicated (E17.5 and postnatal day 56). Increased BL signal (blue) was evident in P56 mice that had been exposed to gestational HFD (F1^mat-HFD^, middle image), as compared to either CD or LPD-exposed animals (F1^mat-CD^, F1^mat-LPD^, left and right, respectively). **b** Abdominal bioluminescence signal was significantly increased in F1^mat-HFD^ offspring (P56) as compared to F1^mat-CD^ or F1^mat-LPD^. BL signal in F1^mat-HFD^ animals was less than that in dietary control animals that inherited the reporter paternally (KI^pat-CD^), suggesting a partial release of silencing. (Number of animals (*N*) indicated in table; Two-way ANOVA on log-transformed data (Diet *p* < 0.0001; Sex *p* = 0.049; Interaction *p* = 0.0046); results of Holm-Šídák’s multiple comparisons follow-up test are shown: ***padj = 0.0004, ****padj < 0.0001, ns=not significant). Source data are provided as a Source Data file. **c** BL signal (blue) in E17.5 embryos from F1^mat-HFD^, F1^mat-LPD^ and F1^mat-CD^ are compared (upper panel) and quantified (lower panel showing Flux levels relative to KI^pat-CD^ controls). BL signals were significantly higher in F1^mat-HFD^ than F1^mat-LPD^ and F1^mat-CD^ embryos. (Number of embryos (*N*) indicated in table; One-way ANOVA on log-transformed data (*p* < 0.0001); results of Dunnett’s multiple comparisons follow-up test comparing to F1^mat-CD^ embryos are shown: ****padj < 0.0001, ns=not significant). Source data are provided as a Source Data file. **d** BL signal (blue) detected ex vivo in organs of male P56 F1^mat-HFD^ animals (left panels: i- liver, ii- white adipose, iii- brain, iv- uterus (taken from female animals), v- testes, vi- brown adipose tissue). Control tissues from P56 F1^mat-CD^ animals (right panels: vii- liver, viii- brain, ix- brown adipose) are shown for comparison.

Foetal exposure to maternal diet low in protein or high in fat, can induce long-lasting changes in gene expression, physiology and behaviour in offspring^10,38–40^. We showed previously that exposure to low protein diet in utero provokes sustained LOI of the maternally expressed imprinted gene *Cdkn1c* in offspring^30^. To examine whether the paternally expressed *Dlk1* gene was sensitive to dietary challenge, we crossed *Dlk1-FLucLacZ* females with wt males and exposed pregnant dams to either control diet (CD), low protein diet (LPD) or high-fat diet (HFD) throughout pregnancy, as outlined in Fig. 3a. Gestational exposure to altered diet resulted in modest changes in dam and embryonic weights (Figs. S3a and S3b), consistent with the dietary model^41–46^. Maternally transmitted *Dlk1-FLucLacZ* is predicted to be silent in offspring derived from these crosses and consistent with this all F1 animals that had been exposed to CD (F1^mat-CD^) in utero showed BL signal at near background levels (Figs. 3a, b). Imprinted expression was also maintained in all F1 animals that had been exposed to LPD (F1^mat-LPD^) in utero. In sharp contrast, exposure to HFD resulted in F1 animals (F1^mat-HFD^) that expressed maternally-derived *Dlk1* (19/21, Figs. 3a, b). This LOI was observed in mature male and female F1^mat-HFD^ offspring, long after gestational exposure and after being switched back to a normal control diet. LOI was also evident in HFD-exposed embryos at E17.5 (Fig. 3c), at a time in gestation where *Dlk1* expression was shown to be exclusively derived from the paternal allele (Fig. 2c). In adults, maternally derived *Dlk1*-reporter signal was evident in dissected brain, liver, thyroid, testes and adipose tissues of F1^mat-HFD^ animals (Fig. 3d, images i-vi), as compared to F1^mat-CD^ (Fig. 3d, images vii-ix). BL signal was generally detected in tissues where paternally-derived *Dlk1* expression would be predicted, although ectopic expression was seen in the uterus.

**Fig. 4:**
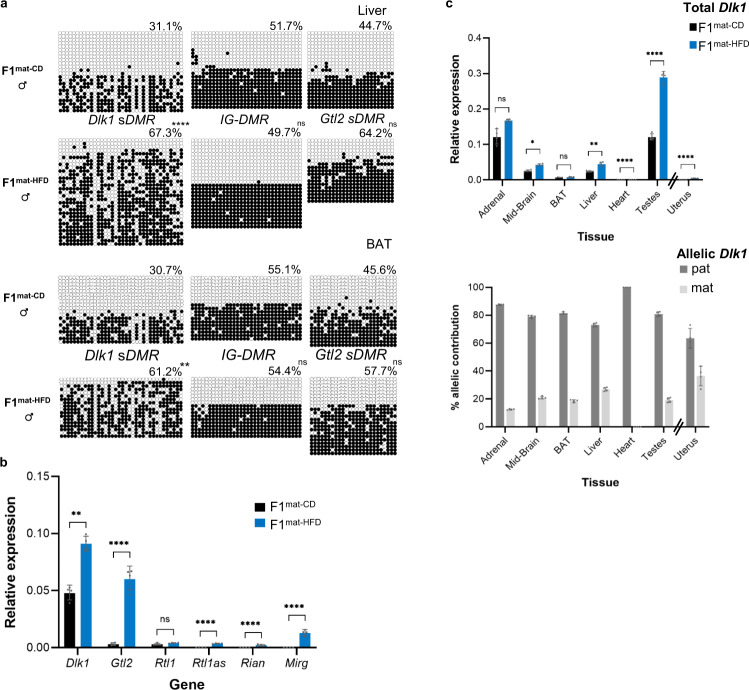
Altered DNA methylation and allelic mis-expression of *Dlk1* in offspring exposed to HFD in utero. **a** DNA bisulphite methylation analysis at *Dlk1 sDMR*, *IG-DMR* and *Gtl2 sDMR* in liver (upper) and brown adipose tissue (BAT) (lower) from representative male P56 F1^mat-CD^ and F1^mat-HFD^ animals. In liver and BAT, hypermethylation was detected at *Dlk1 sDMR*, increased methylation was observed at the *Gtl2 sDMR* (not statistically significant), but *IG-DMR* was unchanged. Closed circles indicate methylated CpGs, open circles un-methylated CpGs. Each row represents an individual clone. Percentages indicate total methylation level of the region from two wt and two KI^mat^ animals. (Kolmogorov-Smirnov test comparing clonal methylation levels, using Holm-Šídák’s correction for multiple comparisons: **padj < 0.0055, ****padj = 6 × 10^−6^, ns=not significant). Source data are provided as a Source Data file. **b** Gene expression (QRT-PCR) at the *Dlk1-Dio3* cluster in the liver of male P56 F1^mat-HFD^ (blue) and F1^mat-CD^ (dark grey) animals. Expression levels for this single tissue comparison were normalised to *β-Actin* expression. (Bars show the geometric mean of relative expression with geometric SD; *N* = 4 + 4 individual mice; unpaired two-sided *t*-tests on delta-Ct values with Holm-Šídák’s correction for multiple comparisons: **padj = 0.0067, ****padj < 0.0001, ns=not significant). Source data are provided as a Source Data file. **c** *Dlk1* expression (QRT-PCR, upper panel) in different tissues from P56 male mice exposed to either control (F1^mat-CD^, black) or high-fat diet (F1^mat-HFD^, blue). Uterus samples from age-matched female mice were also analysed. Expression levels were normalised to *β-Actin*, *18S* and *Hprt*. (Bars show the geometric mean of relative expression with geometric SD; *N* = 4 + 4 individual mice; Two-way ANOVA on delta-Ct values (Tissue *p* < 0.0001, Diet *p* < 0.0001, Interaction *p* < 0.0001); results of Holm-Šídák’s multiple comparisons follow-up test for effect of diet in each tissue are shown: *padj = 0.013, **padj = 0.0042, ****padj < 0.0001, ns=not significant). Allelic *Dlk1* analysis in F1^mat-HFD^ mice (lower panel), using primers that distinguish the reporter from the wt allele, showed a reduced contribution for paternal allele expression (dark grey) when compared to maternal allele expression (light grey). (Bars indicate the mean contribution from each allele ±SD; *N* = 4 + 4 individual mice). Source data are provided as a Source Data file.

*Dlk1* LOI in F1^mat-HFD^ animals was associated with a selective increase in DNA methylation at the *Dlk1 sDMR* (with an increased trend at the *Gtl2 sDMR*, but not the *IG-DMR*) in affected tissues such as liver and BAT (exemplified in Fig. 4a), and with increased gene expression across the entire *Dlk1* cluster (Figs. 4b and S3b). Detailed molecular analyses confirmed significant increases in total *Dlk1* expression in liver, mid-brain and testes of adult F1^mat-HFD^ animals as compared to matched F1^mat-CD^ controls (Fig. 4c, upper panel), while *Dlk1* expression remained extremely low in tissues such as adult heart. Allelic analysis confirmed maternally-derived *Dlk1* expression in adrenal glands, midbrain, BAT, liver, testes and uterus of adult F1^mat-HFD^ animals (Fig. 4c, lower panel), consistent with prior bioluminescent data.

### *Dlk1* misregulation in F2 offspring

**Fig. 5:**
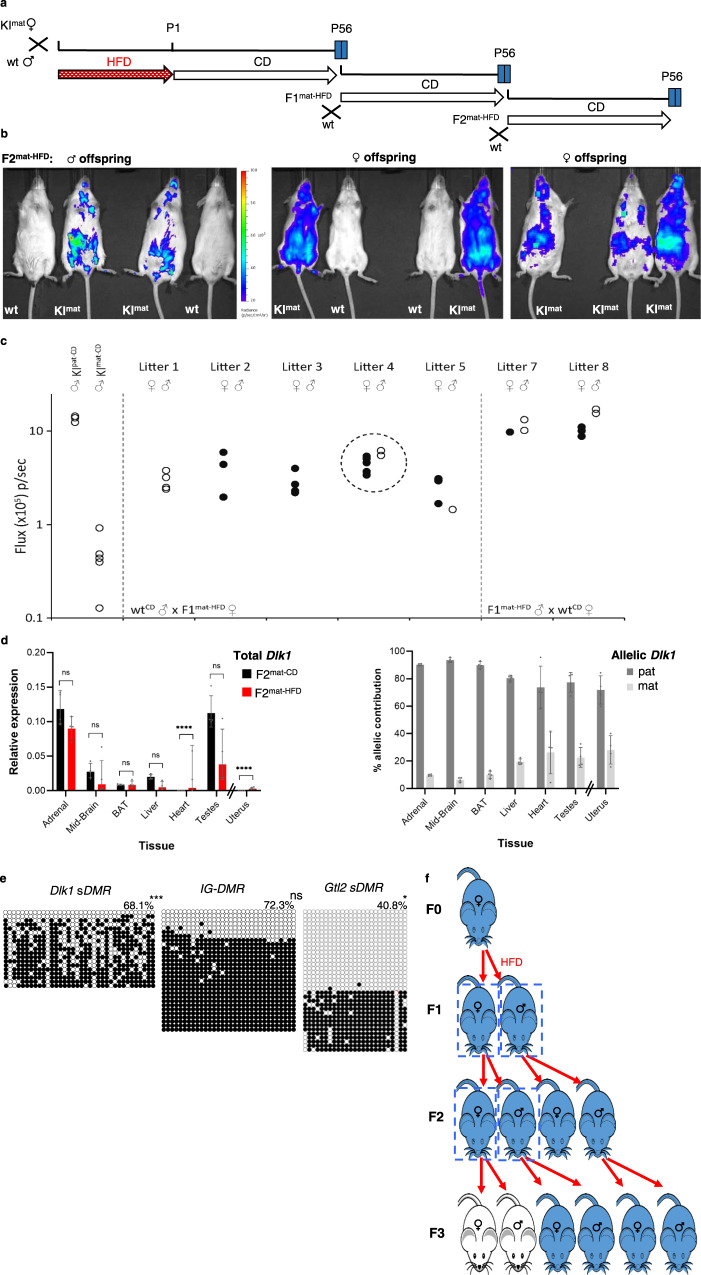
Exposure-induced changes to *Dlk1* imprinting are transmitted to F2 offspring. **a** Schematic for generational studies following HFD exposure. Gestationally exposed animals (*Dlk1-FLucLacZ* F1^mat-HFD^) were bred with wt (CD-fed) mates, maintained on CD, and F2 and F3 offspring examined. **b** BL signal (blue) in F2 offspring (F2^mat-HFD^) derived from F1 HFD-exposed females. Signal was variable and ectopic. **c** Abdominal BL signal in P56 F2^mat-HFD^ males (open-circles) and females (filled-circles), from six F1^mat-HFD^ females and wt^CD^ males (litters 1–5, no litter from female 6) or two F1^mat-HFD^ males and wt^CD^ females (litters 7–8). KI^mat-CD^ and KI^pat-CD^ signal shown for comparison. Litter 4 is represented in (**b**). Source data are provided as a Source Data file. **d** *Dlk1* expression (QRT-PCR, left) in tissues from P56 males (uterus from females) whose mothers were exposed in utero to CD (F2^mat-CD^, black) or HFD (F2^mat-HFD^, red). Expression normalised to *β-Actin*, *18S* and *Hprt* (bars show geometric mean with geometric SD; *N* = 4 + 4 individual mice; Two-way ANOVA on delta-Ct values (Tissue *p* < 0.0001, Diet *p* = 0.002, Interaction *p* < 0.0001); Holm-Šídák’s multiple comparisons follow-up test for diet in each tissue: ****padj < 0.0001, ns=not significant). Allelic *Dlk1* analysis in F2^mat-HFD^ mice (right) showed reduced paternal (dark grey) versus maternal (light grey) expression bias, compared to control conditions (bars indicate mean allelic contribution ±SD; *N* = 4 + 4 individual mice). Source data are provided as a Source Data file. **e** Bisulphite analysis in male P56 F2^mat-HFD^ liver showed *Dlk1 sDMR* hyper-methylation, increased *IG-DMR* methylation (*p*adj = 0.078) and slightly reduced *Gtl2 sDMR* methylation, compared to F1^mat-CD^ (Fig. 4a). Closed circles: methylated CpG, open circles: un-methylated CpG. Rows show individual clones from a representative individual, percentages indicate total methylation from two animals. (Kolmogorov-Smirnov test comparing clonal methylation levels, using Holm-Šídák’s correction for multiple comparisons: *padj = 0.025, ***padj = 0.0001, ns=not significant). Source data are provided as a Source Data file. **f** Summary of altered *Dlk1* expression following gestational HFD. *Dlk1* is silent (white) when transmitted maternally and expressed (blue) when transmitted paternally. Gestational HFD exposure provokes LOI in F1 offspring (blue, box). F1 females transmit altered *Dlk1* expression to F2 offspring (blue, box), whereas F1 males and F2 females transmit *Dlk1* appropriately.

To investigate whether HFD-induced alteration of *Dlk1* was transmitted to subsequent generations, we examined F2 mice derived from crosses between F1^mat-HFD^ females and wt males (F2^mat-HFD^, Fig. 5a). In this setting maternal inheritance is predicted to ensure *Dlk1* silencing, however, BL signal was detected in most F2^mat-HFD^ offspring, as illustrated for a litter of eleven animals (Fig. 5b) in which seven were transgenic (KI^mat^). BL signal distribution suggested that *Dlk1* misexpression was variable among F2^mat-HFD^ animals, consistent with a partial LOI (Fig. 5c). Molecular analyses of liver, heart and mid-brain samples from individual F2^mat-HFD^ mice confirmed heterogeneity in male and female offspring that were derived from F1^mat-HFD^ females and suggested ectopic expression of *Dlk1* in the heart (Figs. 5d and S4a). This was confirmed by immunolabelling of individual heart tissue, with Dlk1 detected in three out of four F2^mat-HFD^ mice, as compared to negative F2^mat-CD^ control tissue (Fig. S4b). Importantly, a strong correlation between elevated levels of Dlk1 detected by molecular and immunohistochemical approaches was noted in individual F2^mat-HFD^ animals (Figs. S4a and S4b). To exclude that *Dlk1* expression showed intrinsic variation between generations, we compared expression in F1 and F2 offspring exposed to CD. As shown in Fig. S4c, *Dlk1* expression in different tissues remained unchanged between generations under CD conditions, and minimal expression was detected in the heart. Together these data support the view that gestational exposure to HFD results in heterogenous and ectopic expression of *Dlk1* in F2 offspring.

Detailed molecular analysis revealed that despite the partial loss of *Dlk1* imprinting seen in F2^mat-HFD^ animals (Fig. 5d, right), overall levels of *Dlk1* expression were generally lower in tissues such as adrenal glands, midbrain, liver and testes, than in controls (Fig. 5d, left). Altered DNA methylation across the *Dlk1-Dio3* locus was also detected in F2^mat-HFD^ somatic tissue, as illustrated in Fig. 5e. In F2 offspring generated from reciprocal crosses (F1^mat-HFD^ males x wt females), *Dlk1* reporter expression was consistent with normal paternal inheritance and imprinting (Fig. 5c, litters 7 and 8).

One explanation for the LOI in second-generation offspring from HFD-exposed females is that in utero exposure affects the developing epigenome of oocytes contained within developing female embryos, as well as affecting F1 somatic tissue. This scenario evokes an intergenerational mode of epigenetic inheritance, rather than requiring trans-generational mechanisms^5^ and predicts that normal imprinting would be restored in subsequent generations (Fig. 5f). Consistent with this possibility, BL signal was undetectable in all F3 transgenic animals derived from LOI-affected F2^mat-HFD^ dams (as exemplified in Fig. S5a), although levels of *Dlk1*-associated gene expression were generally lower in HFD-exposed offspring (F3^mat-HFD^ and F2^mat-HFD^) as compared with CD-exposed (F2^mat-CD^) controls (Fig. S5b).

### Gestational HFD alters transcription in developing oocytes

**Fig. 6:**
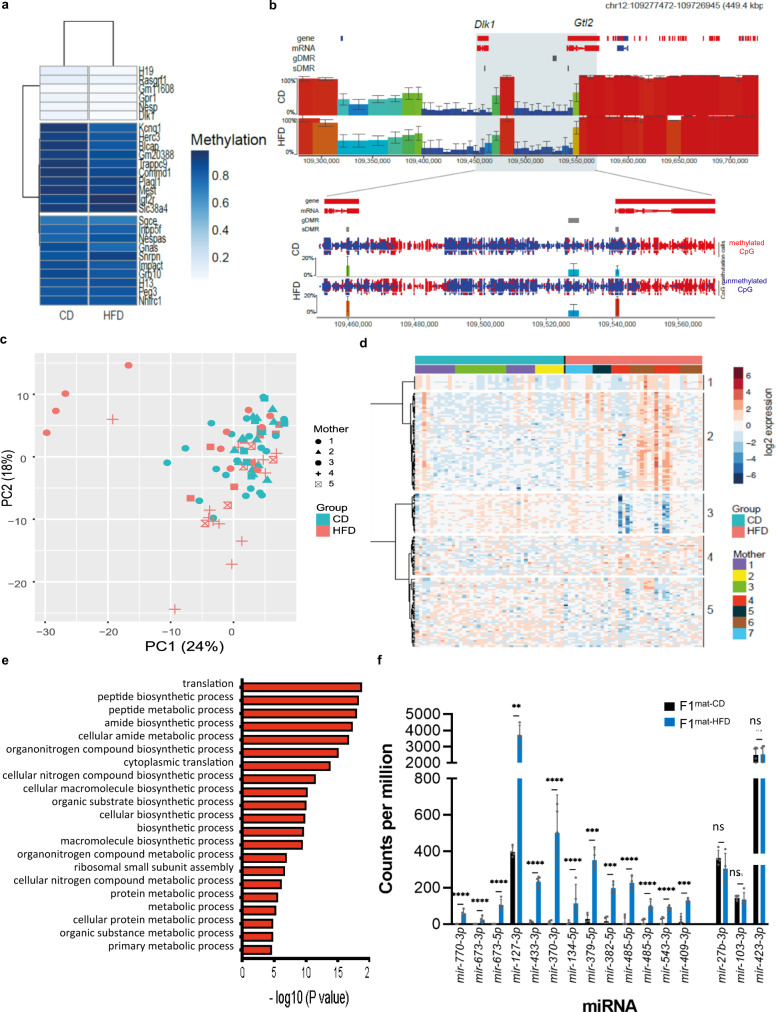
Germline DMRs in single MII oocytes from F1 females are unaffected by dietary exposure but show an altered transcriptional programme. **a** Heatmap representing mean DNA methylation levels for each gametic (g)DMR in F1^mat-CD^ and F1^mat-HFD^ oocytes (merged from 41 and 37 oocyte scBS-seq datasets, respectively). **b** SeqMonk screenshot showing mean DNA methylation in F1^mat-CD^ and F1^mat-HFD^ oocytes over nonoverlapping 100 CpG windows (colour-coded blocks) across a ~450 kb interval encompassing the *Dlk1-Dio3* imprinted cluster with a zoomed-in region (below) showing the CpG methylation calls (methylated red; un-methylated blue) of the *Dlk1-Gtl2* region with quantification over the gDMR and sDMRs. Error bars represent the standard deviation from the mean of 5 pseudo-bulk groupings of 7-8 oocytes each. **c** Principal component analysis of scRNA-seq datasets of individual oocytes from F1^mat-CD^ and F1^mat-HFD^. **d** Heatmap revealing 5 unsupervised clusters of the 166 most variable genes between F1^mat-CD^ and F1^mat-HFD^ oocytes. Top bars identify the F1 donor and diet groups. Clusters 1 to 5 comprised 25, 62, 44, 25 and 9 genes respectively. **e** Major terms highlighted in the gene ontology analysis of up-regulated genes from clusters 1, 2 and 4 (x-axis, -log10 of FDR adjusted *p* values). Gene ontology analysis was performed with GOrilla and summarised with Revigo. **f** Comparison of *Dlk1-Dio3* microRNA (miRs) expression in F1^mat-CD^ (black) and F1^mat-HFD^ (blue) oocytes, alongside three stably expressed miRs (*27b-3p, 103-3p, 423-3p*), analysed by small RNA sequencing. Each of the *Dlk1-Dio3* miRs was found to be significantly more represented in F1^mat-HFD^ oocytes. (Bars represent mean counts per million ±SD; small RNA-seq libraries generated from oocytes from four female mice per group; unpaired two-sided *t*-tests with Holm-Šídák’s correction for multiple comparisons: **padj = 0.0013, ***padj = 0.0005, ****padj < 0.0001, ns=not significant). Source data are provided as a Source Data file.

To better understand the basis of ectopic *Dlk1* reporter expression in F2^mat-HFD^ offspring, we asked whether DNA methylation was perturbed in the gametes of F1^mat-HFD^ mice. As predicted, F1^mat-HFD^ sperm showed DNA methylation exclusively at the *IG-DMR*, with hypo-methylation at *Dlk1 sDMR* and *Gtl2 sDMR* (Fig. S6). F1^mat-HFD^ oocytes were collected individually and processed for parallel genome-wide single-cell bisulphite sequencing and single-cell RNA-seq (scM&Tseq) (quality control shown in Figs. S7a and S7b)^47,48^. In both groups the anticipated bimodal pattern of DNA hypo- and hypermethylated domains in oocytes^48,49^ was retained, with a broadly similar profile assessed over 100-CpG windows (*r* = 0.984) (Figs. S8a and S8b). While differences in methylation were detected in 439 differentially methylated 100-CpG tiles (representing approximately 0.2% of genomic tiles) these were dispersed across the genome (Fig. S8c) and principal component analysis (PCA) indicated no obvious separation of oocytes from different groups (Fig. S8a). Among the imprinted gametic DMRs, high levels of methylation of maternal gDMRs and low levels of methylation of paternal gDMRs were well preserved in F1^mat-HFD^ oocytes (Fig. 6a) and we did not detect significantly increased variation or anomalous gDMR methylation of F1^mat-HFD^ oocytes as compared with F1^mat-CD^ (Figs. S8d and S8e). At the *Dlk1-Dio3* imprinted domain itself, the *IG-DMR* domain remained similarly hypo-methylated in F1^mat-CD^ and F1^mat-HFD^ groups, while the *Dlk1 sDMR* and *Gtl2 sDMR* showed minimal changes in methylation in F1^mat-HFD^ oocytes (Fig. 6b) that were not statistically significant. The lack of changes in DNA methylation levels at the *Dlk1-Dio3* imprinted domain make it unlikely that this is responsible for the highly penetrant maternal transmission of ectopic *Dlk1* expression seen after in utero HFD exposure.

Genome-wide transcription was also examined in these F1^mat-HFD^ oocytes. Although PCA indicated no clear separation of oocytes according to F1^mat-CD^ and F1^mat-HFD^ groupings (Fig. 6c), increased heterogeneity was evident in the HFD group and a subset of 166 genes showed highly variable expression (>0.528 fold from the mean standard deviation) (Fig. S9a and Supplementary Data 1). These genes also showed the largest differences in expression between groups (Fig. S9b). Unsupervised hierarchical clustering segregated the variable genes into five clusters (Fig. 6d), the largest of which (cluster 2) were more likely to be upregulated in HFD oocytes, and were associated with the regulation of translation and modulation of biosynthetic and metabolic processes (Fig. 6e). In contrast downregulated genes did not group together in terms of function (Supplementary Data 1).

**Fig. 7:**
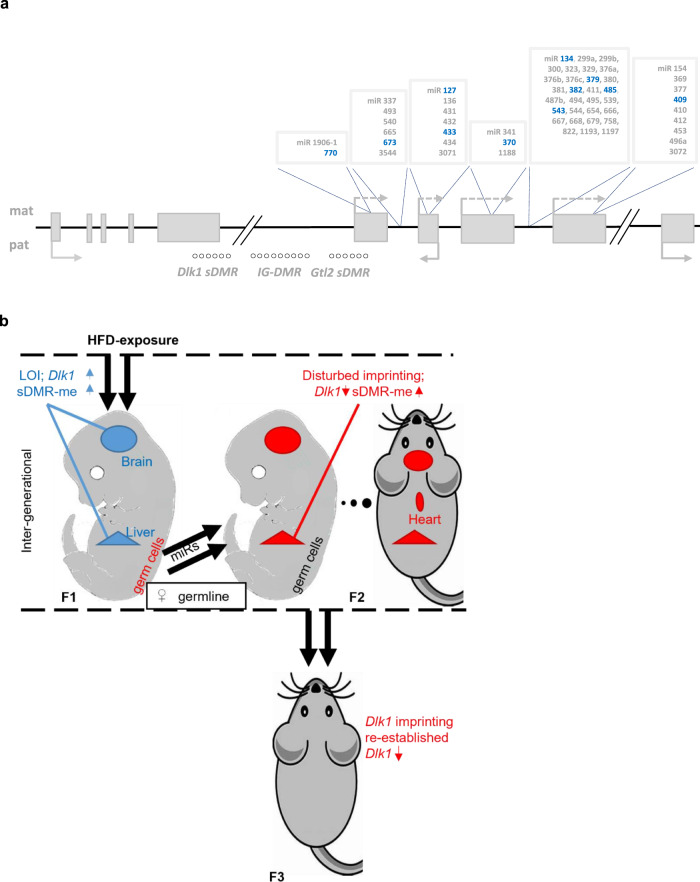
Generational modulation of *Dlk1-Dio3* imprinting in response to HFD exposure. **a** Schematic of *Dlk1-Dio3* cluster miRs that were over-expressed in F1^mat-HFD^ oocytes, as compared to F1^mat-CD^. Over-represented miRs are displayed as blue, while non-expressed miRs are displayed as grey. **b** Schematic summarizing the modifications to *Dlk1* imprinting across generations. Imprinting is disturbed inter-generationally but restored trans-generationally. Blue (increased) and red (decreased) arrows depict expression or methylation levels relative to controls.

These results show that while only limited changes in DNA methylation were seen in oocytes in response to HFD (at a genome-wide level, at the *Dlk1-Dio3* cluster (Fig. 6b), at the imprinted *Ube3a* locus, or a neighbouring non-imprinted locus, *Atp10a* (Fig. S10)), altered transcriptional quality of oocytes from in utero HFD-exposed F1 females was readily detected. In addition, small RNA-sequencing revealed a marked increase in the expression of multiple miR species from within the *Dlk1-Dio3* cluster in F1 oocytes that had been exposed to HFD, as compared to CD (Fig. 6f). The expression of control miR species (*mir 27b-3p*, *mir 103-3p*, *mir 423-3p*) that reside outside the *Dlk1-Dio3* locus, was in contrast, unchanged between F1^mat-HFD^ and F1^mat-CD^ oocyte samples. Importantly, up-regulation of *Dlk1-Dio3* miRs was sustained for long periods after gestational exposure and affected clusters spanning the entire *Gtl2-Rtl1as-Rian-Mirg* domain (Fig. 7a). These data show that miR expression in the developing oocytes is irrevocably altered in response to in utero exposure to HFD and implicate this in the subsequent misregulation of *Dlk1* in F2 progeny.

## Discussion

Our study shows that dietary challenge in pregnancy, and specifically maternal exposure to HFD, induces a loss of *Dlk1* imprinting that impacts two successive generations of offspring. In F1 animals, HFD-exposure provoked a sustained loss of *Dlk1* imprinting in several somatic tissues, a feature that was associated with a corresponding increase in DNA methylation at the *Dlk1 sDMR*. In F2 animals born exclusively to F1-exposed females, *Dlk1* expression was also deregulated and variable *Dlk1* expression was seen among offspring, including ectopic expression in the heart. The vulnerability of F2 offspring born to F1-exposed females inferred that oocytes might be targets of exposure-induced epigenetic change. As female gametogenesis initiates in growing oocytes in the ovary postnatally^50–52^, any direct effect of exposure on de novo DNA methylation appeared very unlikely. Nonetheless exposure to HFD occurs contemporaneously with a widespread DNA methylation erasure in primordial germ cells of the embryo^53^, so we asked whether DNA de-methylation was impaired in F1-exposed oocytes. We saw no evidence of either residual methylation of paternal gDMRs or altered DNA methylation across the *Dlk1-Dio3* domain in these oocytes. Instead, HFD-exposed F1 oocytes showed altered gene expression, detected using genome-wide scRNA-seq, including pronounced changes in expression of many *Dlk1/Gtl2*-associated genes and miRs. This unexpected result suggested that two different epigenetic mechanisms are contributing to *Dlk1* LOI within the developing F1 embryo and these result in distinct temporal-spatial read-outs and impacts (Fig. 7b). Exposure to HFD in utero elicits an inappropriate expression of maternally-derived *Dlk1* in several F1 somatic tissues including BAT, brain and liver, associated with increased DNA methylation at the *Dlk1 sDMR*. HFD-exposure also induced a de-regulated expression of miRs in developing oocytes within female F1 offspring. This is of particular interest as correct expression of *Gtl2*-associated lncRNAs, miRs and snoRNAs is known to be important for restricting the expression of both maternal *Gtl2* and paternal *Dlk1* expression in several other contexts^54–57^. Mis-expression of these RNA species in individual oocytes may therefore underlie the heterogenous misregulation of *Dlk1* expression that we observed in individual F2 offspring, as well as *Dlk1* LOI and de novo ectopic expression of *Dlk1* in cardiac tissues.

The demonstration of altered miR expression in F1 oocytes is surprising given that these were sampled from F1 adults after ovulation and several weeks after HFD exposure. The results implicate an alternative (non-canonical imprinting) route of intergenerational epigenetic transmission where chromatin and miR expression, rather than DNA methylation per se, predispose *Dlk1* and *Gtl2* to deregulation. As histone H3K27me3 is pervasive in the oocyte genome and occupies DNA hypo-methylated domains^58,59^ and has been shown to be responsible for a parallel imprinting mechanism^60,61^, it is tempting to speculate that this modification might play a role. In F1-exposed animals, we demonstrate dramatic increases in *Gtl2*, *Rtl1as*, *Rian* and *Mirg* expression (as well as a plethora of miRs), species that are normally only expressed at low levels and exclusively from the maternal allele^24,27,62^. High levels of transcription across the *Dlk1-Dio3* domain persisted in F1 animals for many months, even after being returned to a normal diet. Collectively these results offer an unanticipated explanation for the deregulation of *Dlk1* seen in the next generation. As expression of miRs across the *Dlk1-Dio3* domain can be self-sustaining^54–57^ and disruption of maternally expressed miRs can alter paternal *Dlk1* expression^57^, constitutive activity across this domain in oocytes would likely result in an imbalance of *Dlk1* expression consistent with what is observed in F2 animals born to HFD-exposed F1 females.

This study examines epigenetic mechanisms that regulate *Dlk1* expression and the intergenerational mechanisms through which gestational exposure to HFD impacts imprinting at the *Dlk1-Dio3* locus. While this focus on imprinting at a single locus can offer access to epigenetic regulatory mechanisms, it is worth noting that imprinting constitutes only a minor fraction of the genome. While the dietary model used here provides fresh information about how imprinting is disrupted and then conveyed to F2 offspring, further studies are needed to examine impacts at non-imprinted sites. Numerous prior studies have shown that gestational exposure to diets low in protein or high in fat impacts gene expression and metabolic circuitry in offspring^9,17,21,30,63^. While it is still unclear how maternal dietary challenges provoke long-lasting changes in offspring; methyl-donor supply, mitochondrial reprogramming, placental stress, and other mechanisms have been inferred^16,38,64–67^. Despite this paucity in understanding, early-life adversities such as this enhance disease risk in offspring^9,41,68^. Genome-wide studies suggest that non-imprinted genes and imprinted genes are equivalently sensitive to dietary challenge^69^, however, relatively minor changes in the expression of imprinted genes are known to provoke major changes in organismal physiology pre- and postbirth^17,70^. Previously we showed that exposure to low protein diet in utero results in an elevated expression of *Cdkn1c* in offspring, through loss of imprinting that begins mid-gestation and is retained into adulthood^30^. In this scenario, LOI was associated with reduced DNA methylation at the *Cdkn1c sDMR* and was partially rescued by folate supplementation. Although LPD-induced *Cdkn1c* LOI resulted in altered offspring behavior^40,71^, no obvious phenotypic changes or LOI was evident in F2 mice, in marked contrast to HFD-induced LOI observed here for *Dlk1*.

Several reports link maternal overnutrition to phenotypic changes in mice that span three or more generations, which are mediated through the germline, and would therefore be viewed as transgenerational^63,72,73^. These published studies have implicated sperm RNAs, miRs and tRNA-derived small RNAs in the transmission of epigenetic memory. Here we show that exposure to HFD in utero results in intergenerational epigenetic changes that are mediated through the female germline and provoke heterogenous misexpression of *Dlk1* in F2 mice. As our work suggests that *Dlk1* imprinting is re-instated in the subsequent F3 generation, future studies will be required to determine any transgenerational impact. Irrespective of this outcome, our study raises the intriguing possibility that genomic imprinting mechanisms that harness multiple layers of epigenetic control can enable the phenotypes of successive generations of offspring to be modified in response to environmental challenges that are precociously sampled ahead of birth.

## Methods

### Generation of targeted ESCs and mice

The *Dlk1-FLucLacZ* (B6NTac) line was created by Taconic Biosciences and ESCs and animal founders were delivered to Imperial College. A *firefly luciferase* and *LacZ* were knocked in to the 3’UTR of the *Dlk1* gene, with T2A sites. This approach produces a single RNA, under the control of the *Dlk1* promoter, in a non-disruptive manner. Upon translation the T2A sites self-cleave, liberating independent proteins of FLuc, β-galactosidase and Dlk1. Mice were back-crossed onto a B6(Cg)-*Tyr*^*c-2J*^/J (Jackson Labs, C57Bl/6 J albino) background for >6 generations.

### Maintenance of mice

Mice were handled and all in vivo studies were performed in accordance with the United Kingdom Animals (Scientific Procedures) Act (1986). Mouse work was approved by the Imperial College AWERB committee and performed under a UK Home Office project license. Mice were housed on a 12-h light-dark cycle with a temperature range of 21 + /− 2 °C and a humidity range of 55 + /− 10% in pathogen-free conditions. The *Dlk1-FLucLacZ* line was maintained on a B6(Cg)-*Tyr*^*c-2J*^/J (Jackson Labs, C57Bl/6 J albino) background. For mating, males were set up with not more than three females and morning plug checking was performed. Upon plug discovery, females were considered E0.5.

### Genotyping of animals

Genomic DNA was isolated from 4-week old ear biopsies or embryonic tails by digestion in lysis buffer (0.05 M Tris HCl pH 8, 0.025 M EDTA, 0.031% SDS, 0.02 M NaCl, 80 μg/ml Proteinase K (Sigma-Aldrich)) at 50 °C with rocking. DNA was diluted 1:2 in 10 mM Tris HCl pH8 and 1 µl of diluted DNA was used in PCR analysis (primer sequences provided below).

### Diet studies

*Dlk1-FLucLacZ* females were set up with B6(Cg)-*Tyr*^*c-2J*^/J males and upon vaginal plug discovery, matings were separated. Females were fed either a low protein chow (5769, TestDiet), a control chow (5755, TestDiet) or a high-fat chow (45% energy from fat, 58V8, TestDiet). All animals were returned to the control diet at E18.5. Pregnant dams and embryos were imaged at E17.5 and offspring were imaged at P56. For multi-generational studies, *Dlk1-FLucLacZ* (males and females) mice that had been exposed to in utero HFD for the duration of pregnancy were aged to 10 weeks, with access to control diet *ad libitum* and set up with a B6(Cg)-*Tyr*^*c-2J*^/J partner. Offspring were aged to P56 prior to analysis.

### Bioluminescent imaging

D-Luciferin (Perkin Elmer) was dissolved in H_2_0 at 30 mg/ml. Mice were weighed and injected IP with 0.15 mg/g body weight, before being anaesthetized with isofluorane. Mice were imaged 10 min postinjection, in an IVIS Spectrum (Perkin Elmer) under anaesthesia. Images of adult mice and pregnant dams were taken at field of view (FOV) C, with binning 4 and 180 s exposure. For imaging of embryos, pregnant females were injected with D-Luciferin at least 12 min prior to imaging. Embryos were dissected into 24 well dishes containing PBS and placed in the IVIS Spectrum. Images of embryos were taken at FOV A, with binning 4, focus 1 cm and 180 s exposure. No additional D-Luciferin was added, and imaging continued for up to 35 min postinjection. For ex vivo imaging of tissue, mice were culled at least 10 min after D-Luciferin injection, organs were removed and placed in clean dishes containing PBS. No further D-Luciferin was added to samples. Analysis of images was performed on Living Image software 4.5 (Caliper Life Sciences). For quantification of bioluminescent signal, regions of interest were drawn around the specified region and signal flux within the region was calculated.

### LacZ staining

Tissues or embryos were dissected and placed in cold LacZ fixative (2% formaldehyde, 0.2% glutaraldehyde, 0.02% Nonidet P-40, 1 mM MgCl_2_, 0.1 mg/ml Sodium Deoxycholate in PBS) for 4 hr or O/N, at 4 °C with rocking. The tissue was washed in PBS, embedded in OCT (Fisher Scientific) and stored at −80 °C. Embedded tissues were sectioned (15 µm) with a CM1950 cryostat (Leica). Embryos and sectioned tissues were subsequently washed in PBS before being placed in LacZ stain (0.4 mg/ml X-Gal, 4 mM Potassium Ferrocyanide, 4 mM Potassium Ferricyanide, 1 mM MgCl_2_, 0.02% Nonidet P-40 in PBS) for 4–6 (embryos) or 24–48 (sectioned tissue) hr at 4 °C (embryos) or RT (sectioned tissue) with gentle rocking. Upon completion, embryos were washed twice in PBS before transfer to 70% ethanol and storage at 4 °C. Sectioned tissues were washed twice in PBS and mounted with coverslips and Fluoroshield containing DAPI (Insight Biotechnology). Slides were imaged with NanoZoomer-XR (Hamamatsu).

### Optical projection tomography

LacZ stained E11.5 embryos (as above) were mounted in 2% low melting point agarose cylinders, dehydrated through graded methanol solutions and maintained in 100% methanol. For optical clearing, samples were immersed overnight in BABB (1:2 Benzyl benzoate: Benzyl alcohol, Sigma Aldrich). Optical projection tomography (OPT) was performed on an in-house low-magnification imaging system^30,74^. Briefly, cleared samples were suspended from a rotation stage (T-NM17A200, Zaber Technologies Inc) in a BABB-filled cuvette. Images were acquired with a CCD camera with 2 × 2 pixel binning (Clara, Andor Technology Ltd) using a telecentric zoom lens (modules NT56-625, NT59-671 and NT59-672, Edmund Optics Ltd). For LacZ stain imaging, transmitted light images were acquired through a 716 ± 20 nm band-pass filter (FF01-716/40-25, Laser 2000 UK Ltd) every 1° over a full 360° rotation. Average illumination and background images were also acquired, and these images were combined to form an integrated absorption coefficient image at each projection angle. 3D reconstructions of the absorption coefficient per voxel were produced using a filtered back-projection algorithm^75^. The whole sample volume was reconstructed from fluorescence OPT imaging with 473 nm excitation (Cobolt BluesTM, Cobolt AB) and acquisition at 520 ± 17 nm (FF01520/35-25, Laser 2000 UK Ltd).

### Immunostaining

The tissue was dissected and fixed in ice-cold 4% PFA in PBS O/N with gentle rocking. The tissue was washed in PBS the following morning, embedded in OCT (Fisher Scientific) and stored at −80 °C. Embedded tissues were sectioned (12 µm) with a CM1950 cryostat (Leica). Sections were blocked with 10% normal goat serum (Thermo Scientific) in PBS, 0.1% Tween20 at RT for 1 h. Following blocking, samples were incubated with primary antibodies (antibody details provided below) in blocking buffer for 1 hr at RT. Samples were washed 3X in PBS, 0.1% Tween 20 for 5 mins at RT. Samples for immunofluorescence were incubated with fluorescently tagged-secondary antibodies in blocking buffer for 1 hr at RT. Samples were washed 3X in PBS, 0.1% Tween 20 for 5 mins at RT and mounted with coverslips and Fluoroshield containing DAPI (Insight Biotechnology). Images were taken with identical gain and exposure times in all instances, using a LSM880 confocal microscope (Leica). Immunofluorescence images were analysed with Zen Blue 3.4 (Zeiss). Samples for immunohistochemistry were incubated with an HRP-conjugated secondary antibody in blocking buffer for 1 h at RT. Samples were washed 3X in PBS, 0.1% Tween 20 for 5 min at RT. Samples were incubated with DAB substrate (ab64238, Abcam) for 10 min and washed 2X in PBS, 0.1% Tween 20 for 5 min at RT. Samples were counter-stained with Haematoxylin solution (Merck) for 30 s, with immediate washing 3X in PBS, 0.1% Tween 20 for 5 min at RT. Finally, samples were mounted with coverslips in mounting medium (Insight Biotechnology). Slides were imaged with NanoZoomer-XR (Hamamatsu). Immunohistochemistry images were analysed with NDP.view2 (Hamamatsu).

### RNA extraction and QRT-PCR analysis

RNA was extracted with TRIzol (Thermo Scientific) according to manufacturer’s protocol and all RNA precipitation steps were performed with 100% ethanol. Reverse transcription was performed using Superscript III Reverse transcriptase (Invitrogen) as per the manufacturer’s protocol, with minor modifications. RT-PCR was performed on a CFX96 Real-Time System (Bio-Rad) with QuantiTect SYBR Green Master Mix (Qiagen) as per the manufacturer’s protocol, with each well pipetted in technical triplicate and each plate run in technical duplicate.

Average Ct values for each biological replicate (each *N*) were generated from the technical replicates. For relative expression, ΔCt values were calculated relative to *β-Actin* for comparisons within a single tissue, or using the mean of three housekeeping genes (*β-Actin, 18S, Hprt*) for multi-tissue comparisons. ΔCt values (logarithmic data, normally distributed) were used for statistical analyses, with details provided in the figure legends. The geometric means of antilog values were plotted, with error bars indicating the geometric SD. For allelic contributions, ΔCt values were calculated as ΔCt = Ct(KI^mat^) – Ct(KI^pat^), and converted to percentage contributions: KI^pat^ % = 100/(2^ΔCt^ – 1); KI^mat^ % = 100/(2^−ΔCt^ – 1). Primer sequences are provided below.

### Preparation of MII-oocytes

Superovulation of 6-week old females was performed by evening IP injection of pregnant mare serum (PMS) followed by injection of human chorionic gonadotropin (HCG) 48 h later. The following morning, mice were sacrificed via cervical dislocation and cumulus oophorus complexes (COCs) were collected from the oviduct via mechanical dissection. After digestion with hyaluronidase (Sigma Aldrich), MII oocytes were washed in sterile PBS and collected in RLT buffer (Qiagen). Oocytes for the single-cell experiments were stored in 96-well plates at −80 °C until further processed, while oocytes for small RNA experiments were processed immediately.

### Single-cell bisulphite and RNA-sequencing of MII-oocytes

Cell lysis was performed and Poly-A RNA was captured using oligo-dT conjugated to magnetic beads. Single-cell (sc)RNA-seq libraries were prepared according to the G&T-seq and Smartseq2 protocol^76^. The lysate containing gDNA was purified on AMPureXP beads before bisulphite-sequencing (BS-seq) libraries were prepared according to the scBS-seq protocol that has previously been described in detail^77^. Libraries were sequenced on a NextSeq500 (Illumina) with HighOutput 75 bp Paired-End (scBS-seq) or Single End (scRNA-seq) sequencing.

For scBS-seq analysis, read alignment to the GRCm38 reference genome, deduplication and methylation calling was performed using Bismark v0.22.1^78^. Oocytes with a global methylation greater than 50%, or X chromosome CGI methylation greater than 16%, were discarded from analysis. Methylation calls from all oocytes of the same group were pooled to obtain DNA methylation levels at 19 maternal gDMRs, three paternal gDMRs and two secondary DMRs. These DNA methylation levels represent the average across all CpG sites within the DMR. DMR identification from single-cell BS-sequencing was performed by pseudobulking the single oocyte datasets in groups of nine cells and quantifying DNA methylation over 100-CpG tiles to overcome the sparsity in single-oocyte data. Differential methylation was tested using a weighted logistic regression. This procedure was repeated 100 times shuffling the members of the pseudobulk groupings to avoid quantification biases. A DMR was called if the observed methylation difference was at least 10% and had an FDR < 0.05 in at least 70 of the 100 shuffling events^79^.

For scRNA-seq analysis, reads were mapped with hisat2 v2.1.0^80^ against the GRCm38 reference genome and gene expression was quantified over the mouse oocyte transcriptome as log2-transformed reads per million using SeqMonk v1.46.0 (https://www.bioinformatics.babraham.ac.uk/projects/seqmonk/). Principal component analysis was performed using R (R Core Team, 2016). The top variable genes were identified using the function variation plot in SeqMonk with Standard Error of the Mean parameters. Hierarchical clustering of the expression levels of the most variable genes was performed using R (R Core Team, 2016). GO analysis was done using the Gene Ontology enRIchment anaLysis and visuaLizAtion tool (GOrilla) (http://cbl-gorilla.cs.technion.ac.il/)^81^, followed by reduction of terms with Revigo [http://revigo.irb.hr]^82^.

### miR analysis of MII-oocytes

Total RNA was extracted from bulk oocytes with TRIzol (Thermo Scientific) according to the manufacturer’s protocol. Small RNA library preparation was performed with NEBNext Small RNA Library Prep (NEB), and microRNAs were isolated by size selection on 6% TBE PAGE gels (Novex) with clean up performed using Monarch PCR and DNA clean up kit (NEB), according to the manufacturer’s protocol. Sample validation was performed on a Bioanalyzer 2100 using High Sensitivity DNA analysis kit (both Agilent) and libraries were sequenced on a MiSeq (Illumina) and analysed using sRNAtoolbox (https://arn.ugr.es/srnatoolbox/srnabench/)^83^.

### Clonal bisulphite sequencing (tissue)

Bisulphite modification of DNA was carried out with the EZ Gold DNA Methylation Kit (Zymo Genetics) according to the manufacturer’s recommendations. PCR primers (sequences provided below) that specifically recognize bisulphite-converted DNA were used to amplify regions spanning the three imprinted DMRs associated with the *Dlk1-Dio3* imprinted region, with TaKaRa EpiTaq™ HS (Takara), using the manufacturer’s protocol. PCR products were separated on an agarose gel and bands corresponding to the predicted size were excised and cleaned up with a Gel Extraction kit (QIAquick, Qiagen). Ligation of product was performed using Clone JET PCR cloning kit (Thermo Scientific) as per the manufacturer’s protocol, before transformation into DH5α cells. Bacteria were plated onto LB/Ampicillin plates and grown up overnight at 37 °C. Colonies were picked (normally 24 per sample) and expanded in LB/Ampicillin broth overnight at 37 °C. The following morning, plasmids were purified with the Wizard® SV 96 Plasmid DNA Purification System (Promega) according to the manufacturer’s protocol and sent for Sanger Sequencing (GeneWiz).

### Pyrosequencing (bisulphite, tissue)

DNA (0.5–1 µg) was bisulphite treated using the two-step protocol of the Imprint DNA Modification Kit (Sigma). The *Dlk1 sDMR* region was amplified from bisulphite converted DNA through PCR with HotStarTaq DNA Polymerase (Qiagen) (primer sequences provided below). PCR products were shaken at 1,400 rpm with Streptavidin Sepharose High Performance beads (GE healthcare) in binding buffer (10 mM Tris-HCl pH7.6, 2 M NaCl, 1 mM EDTA, 0.1% Tween-20) for 20 min. The biotinylated strand of the product was purified using the PyroMark Q96 Vacuum Workstation (Qiagen). The sequencing primer was annealed to the template in annealing buffer (20 mM Tris-acetate pH7.6, 2 M magnesium acetate) at 85 °C for 4 min. Sequencing was performed with the PyroMark Q96 MD pyrosequencer (Qiagen) using PyroMark Gold Q96 Reagents (Qiagen).

### Bisulphite primers

Clonal

*Dlk1 sDMR* F: CCCCATCTAACTAATAACTTACA

*Dlk1 sDMR* R: GTGTTTAGTATTATTAGGTTGGTG

*IG-DMR* F: GTATGTGTATAGAGATATGTTTATATGGTA

*IG-DMR* R: GCTCCATAACAAAATAATACAACCCTTCC

*Gtl2 sDMR* F: GAAGAATTTTTTATTTGGTGAGTGG

*Gtl2* *sDMR* R: CAACACTCAAATCACCCCCC

Sequencing R: CAGGAAACAGCTATGAC

Pyrosequencing

*Dlk1 sDMR* F: GTTAGAAAGGGGGTATTTGTTTTTAGTAT

*Dlk1* *sDMR* R: 5’[Btn]CTTTCATAAACACCTTCAAAATATATTACT

Sequencing F: ATTTGTTTTTAGTATATTTAGGTGA

### QRT-PCR Primers

*Dlk1* F: GAAAGGACTGCCAGCACAA

*Dlk1 Total* R: CACAGAAGTTGCCTGAGAA

*Dlk1 tg* R: GCCGGGCCTTTCTTTATGTT

*Dlk1 wt* R: CCCCGGTAATAGAGAAGGGC

*B-Actin* F: CCTGTATGCCTCTGGTCGTA

*B-Actin* R: CCATCTCCTGCTCGAAGTCT

*Gtl2* F: CGAGGACTTCACGCACAAC

*Gtl2* R: TTACAGTTGGAGGGTCCTGG

*Rtl1* F: TACTGCTCTTGGTGAGAGTGGACCC

*Rtl1* R: GGAGCCACTTCATGCCTAAGACGA

*Rtl1as* F: TCTCCACTCGAGGGTACTCCACCT

*Rtl1as* R: GTGGAGAACTTCGCTGTCATCGC

*Rian* F: ATGTCTGCTGCCCTGTCGTCT

*Rian* R: GCGGTCACTGCCAAGGTCTCT

*Mirg* F: GTTGTCTGTGATGAGTTCGC

*Mirg* R: GTTCTTGAACATCCGCTCC

*18S*F: CCTGGATACCGCAGCTAGGA

*18S*R: GCGGCGCAATACGAATGCCCC

*Hprt* F: AGTGTTGGATACAGGCCAGAC

*Hprt* R: CGTGATTCAAATCCCTGAAGT

### Genotyping primers

*Dlk1* Geno F: AGTTTGCAAGCTGCACTTGG

*Dlk1* Geno R: CTTTGGAGCTAGATCTTTCAGTGG

### Antibodies

Anti-Dlk1 [3A10]: ab119930 (Abcam) Mouse monoclonal. 1:100 dilution.

Antifirefly Luciferase [EPR17790]: ab185924 (Abcam) Rabbit monoclonal. 1:100 dilution.

Goat antirabbit 568: A-11011 (Thermo Scientific). 1:1000 dilution.

Goat antimouse 488: A-10680 (Thermo Scientific). 1:1000 dilution.

Goat antimouse HRP-conjugated. #31430 (Thermo Scientific). 1:1000 dilution.

### Calculations, graphs and statistical analysis

Microsoft Excel and GraphPad Prism (v9.2.0) were used for calculations, statistical analysis and the preparation of graphs. In general, figures show the mean and standard deviation or geometric mean and geometric standard deviation, with details provided in the figure legends. Data were tested for normality prior to statistical tests where relevant (D’Agostino-Pearson omnibus K2), and statistical tests were performed on log-transformed data (delta-Ct values for QRT-PCR data) where appropriate, as indicated in the figure legends. Multi-group comparisons were generally tested by One-way or Two-Way ANOVA, followed by either Dunnett’s (comparisons to control only) or Holm-Šídák’s (comparisons between each group) two-sided multiple comparisons follow-up tests. For experiments where pair-wise comparisons were determined a priori, unpaired two-sided t-tests with Holm-Šídák’s correction for multiple comparisons were performed directly. DNA bisulphite data were compared using the Kolmogorov-Smirnov test (comparing clonal methylation levels), using Holm-Šídák’s correction for multiple comparisons. Specific details of statistical tests and results are provided in the figure legends. Raw data and output from statistical analyses are provided in the Source Data file.

### Reporting summary

Further information on research design is available in the Nature Research Reporting Summary linked to this article.

## Supplementary information

Supplementary Information

Description of additional Supplementary Files

Supplementary Movie 1

Supplementary Movie 2

Supplementary Movie 3

Supplementary Data 1

Reporting Summary

## Data Availability

The data that support this study are available from the corresponding author upon reasonable request. Single cell sequencing data generated in this study have been deposited in the GEO database under accession code GSE175538. Source data are provided with this paper.

## Source data

Source Data
