# Supplementary material for: Epigenetic changes induced by in utero dietary challenge result in phenotypic variability in successive generations of mice
Source: Nat Commun. 2022 May 5;13:2464. doi: 10.1038/s41467-022-30022-2 (PMC9072353; doi:10.1038/s41467-022-30022-2)
Supplement: Supplementary file 1 — Supplementary Information [file 41467_2022_30022_MOESM1_ESM.pdf]

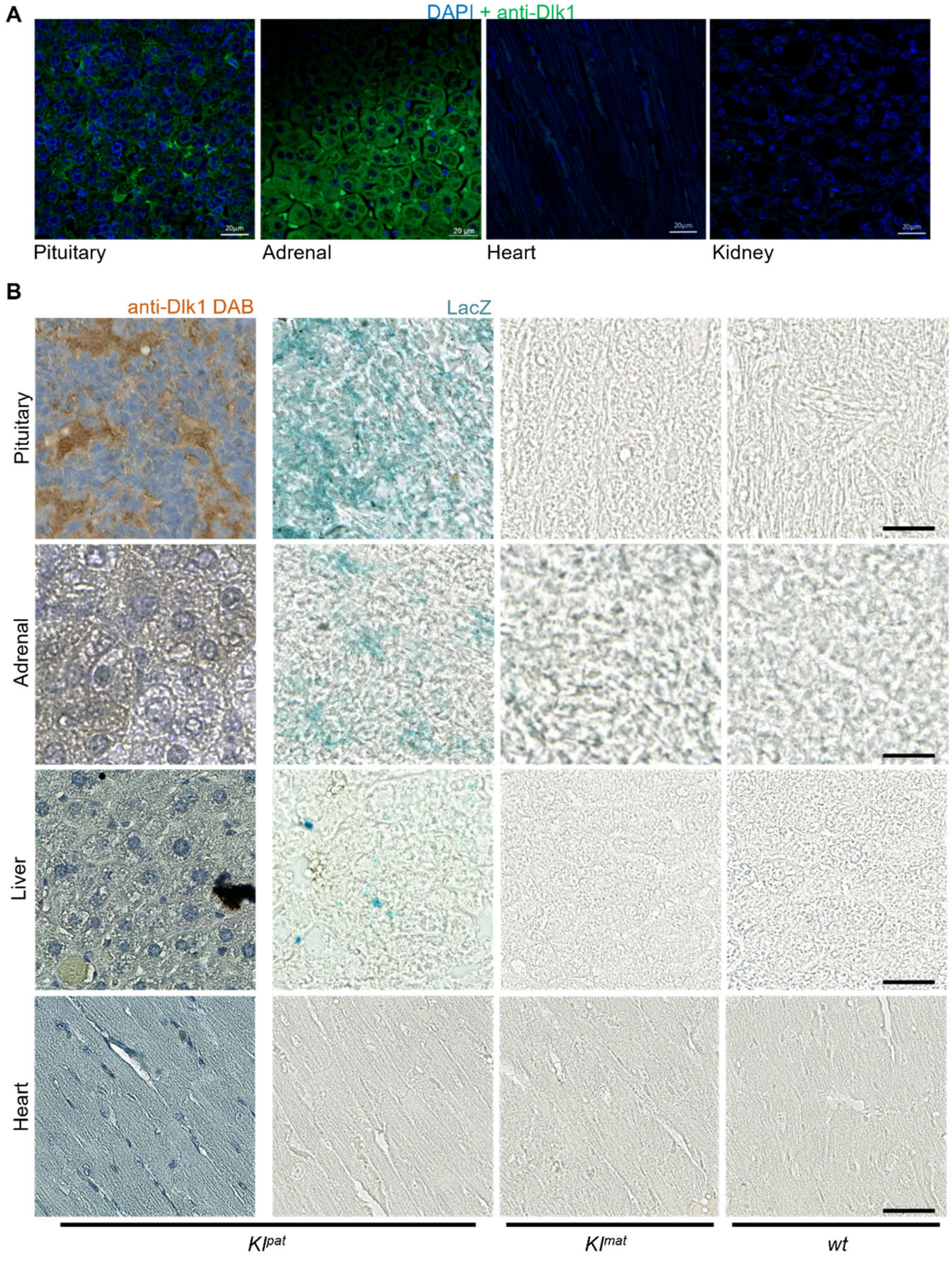

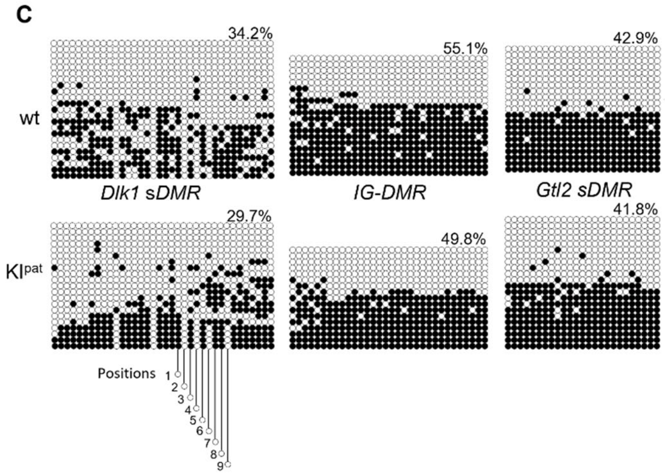

D

Pos	Average Methylation					
	wt	SD	KI^{pat}	SD	KI^{mat}	SD
**1**	32.99	7.85	34.63	1.19	33.66	1.61
**2**	53.37	4.47	56.20	9.52	52.32	9.57
**3**	37.74	5.11	41.19	6.73	40.93	2.14
**4**	47.68	8.57	49.39	10.63	48.10	7.20
**5**	40.36	1.43	41.53	3.67	41.96	0.61
**6**	55.20	7.93	58.62	10.85	58.54	8.56
**7**	44.94	5.78	49.51	9.39	47.61	3.92
**8**	59.41	1.27	59.65	8.63	58.99	2.75
**9**	62.33	9.78	65.19	14.42	62.98	13.89

Figure S1. Imprinted tissue-specific expression of Dlk1 in *Dlk1-FlucLacZ* reporter mice

A. Representative images of anti-Dlk1 immunolabeling (green) of wild type (wt) adult male mouse tissues revealed abundant Dlk1 protein within cells in pituitary and adrenal glands, but not in heart and kidney. Nuclei counterstained with DAPI (blue), scale bar=20 µm. Tissue from three animals was sectioned, with staining performed twice for each.

B. Representative images of tissue sections from *Dlk1-FLucLacZ* and wt adult males, labelled to reveal Dlk1 protein (DAB; brown, left column) or stained for β-galactosidase activity/LacZ (blue, right-hand columns). In KI^{pat} mice, Dlk1 was detected in pituitary and adrenal glands with low level expression in liver, and minimal (background) detection in heart. LacZ staining of sequential KI^{pat} tissue sections showed a very similar distribution with positive cells detected in pituitary, adrenal and liver, but not heart tissue. LacZ positive cells were not detected in age-matched tissues from KI^{mat} or wt controls. DAB preparations were counter-stained with haematoxylin (dark blue). Scale bars=20 µm. Tissue from four animals per group was sectioned, with staining performed twice (DAB) or thrice (LacZ) for each.

C. Clonal bisulphite methylation analysis at *Dlk1 sDMR*, *IG-DMR* and *Gtl2 sDMR* in liver of male P56 mice. Closed circles indicate methylated CpGs, open circles un-methylated CpGs. Each row represents an individual clone. Percentages indicate total methylation of the region from two wt and two KI^{pat} animals, with clones shown for representative individuals. Methylation patterns of all three DMRs that control *Dlk1-Dio3* imprinting were unchanged between wt and KI^{pat} P56 adult mice (Kolmogorov-Smirnov test comparing clonal methylation levels, using Holm-Šídák's correction for multiple comparisons). Positions 1-9 represent CpGs subsequently analysed by pyrosequencing (Figure S1D). Source data are provided as a Source Data file.

D. *Dlk1 sDMR* methylation levels were analysed by pyrosequencing, with no differences between wt, KI^{pat} or KI^{mat}, at any of the CpGs analysed (Holm-Šídák's two-sided multiple comparisons test). Samples used were identical to those used in the clonal analysis (Figure S1C). CpG position corresponds to those identified in Figure S1C. (N=2+2+2 individual mice). Source data are provided as a Source Data file.

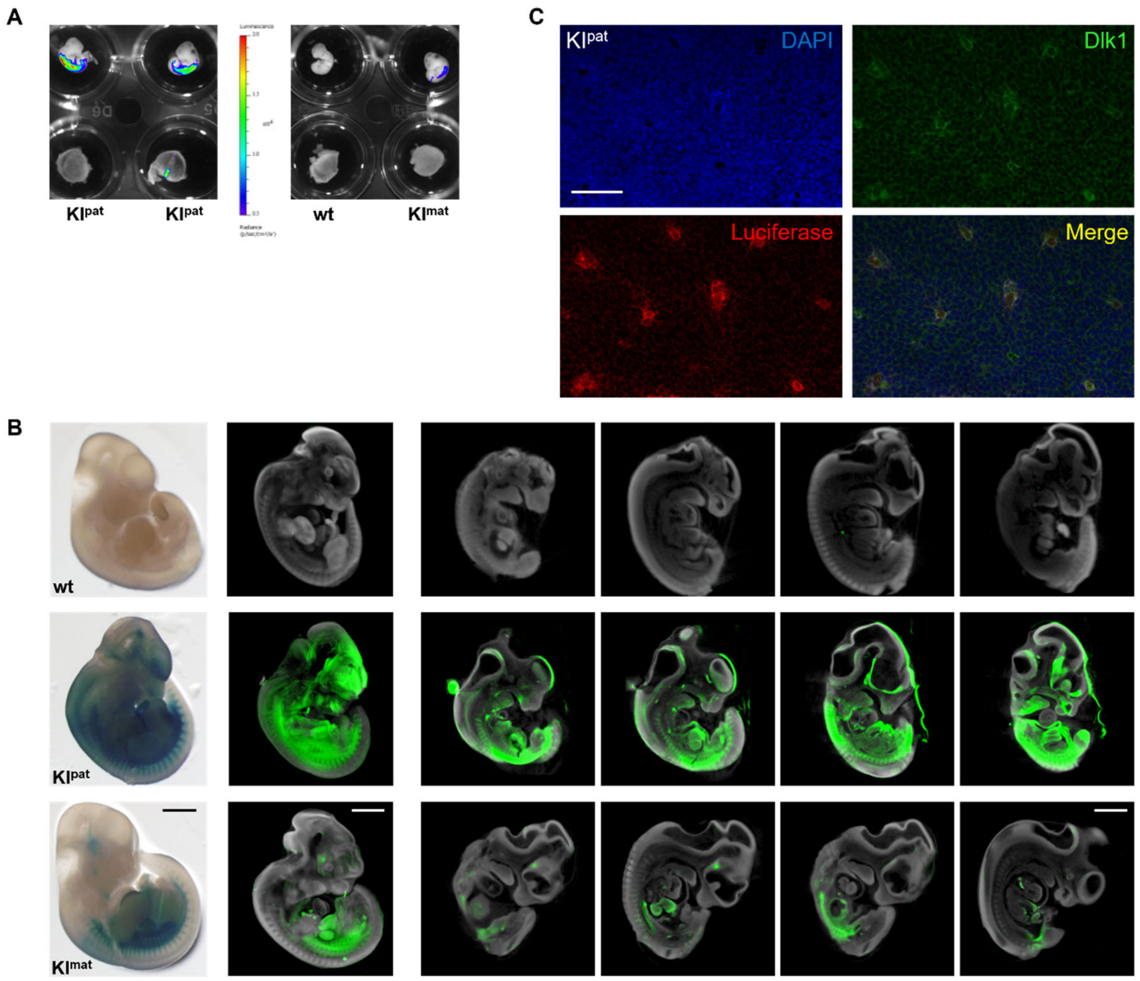

Figure S2. Expression of *Dlk1* maternal and paternal alleles in E11.5 embryos

A. BL imaging of *Dlk1-FLucLacZ* embryos at E11.5, where luciferase activity (blue) was seen in head and abdomen of both KI^{pat} and KI^{mat} (stronger in KI^{pat}) embryos. These images are an un-cropped image of those used in Figure 2C, with corresponding placentas shown below each embryo.
B. LacZ staining of *Dlk1-FLucLacZ* whole embryos at E11.5 (left column) showing labelling of cartilage, brain and abdomen upon paternal inheritance of the reporter, with limited staining detectable upon maternal inheritance and no staining detected in wt embryos. Scale bar: 2 mm. Optical Projection Tomography (OPT) of LacZ stained E11.5 *Dlk1-FLucLacZ* embryos showing whole embryos (inner left column) and individual optical slices (right columns). Absorbance (green) was measured in the liver, cartilage, gonadal ridges and a subset of forebrain regions in KI^{pat} embryos. Absorbance was weaker in KI^{mat} embryos with restricted expression that overlaps with a subset of expression sites in KI^{pat} embryos. Minimal signal was detected in wt embryos. Scale bars represent 2 mm. Staining was performed on at least four embryos per genotype, coming from two litters, with representative images shown. 3D OPT data can be viewed in Supplementary Movies 1-3.
C. Immunostaining of Dlk1 (green, upper right) and Luciferase (red, lower) expression in the E11.5 brain, with nuclear DAPI staining (blue, upper left). Overlapping Dlk1 and Luciferase expression was observed in KI^{pat} tissue (merge, lower right). Scale bar represents 50 μm. Sections were generated from four embryos, and staining was performed twice for each; representative images are shown.

A

Diet	N	Dam weight g
CD	8	29.2 ± 1.5
HFD	7	31.6 ± 2.3*
LPD	2	26.75^{NT}

B

Diet	N	E17.5 weight mg
CD	9	803.5 ± 18.7
HFD	12	778.3 ± 30.4^{ns}
LPD	8	753.8 ± 24.1***

C

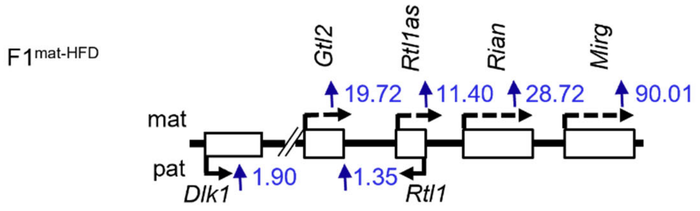

Figure S3. ***In utero*** **exposure alters** ***Dlk1-Dio3*** **gene expression in F1 offspring**

A. Dam weights at E18.5 following gestational exposure to HFD, LPD or CD. A significant increase in weight was observed following HFD exposure, when compared to CD. (Number of dams weighed (N) indicated in table; Student's unpaired two-tailed t-test, *p=0.029, NT=not tested). Source data are provided as a Source Data file.
B. E17.5 embryonic weights following dietary exposure to maternal HFD, LPD or CD. LPD exposed embryos were found to weigh significantly less than CD exposed embryos. A small difference was observed between HFD and CD, but this did not achieve significance. (Number of embryos weighed (N) indicated in table; One-way ANOVA (p=0.002); results of Dunnett's multiple comparisons follow-up test comparing to CD embryos are shown: ***padj=0.0009, ns=not significant (padj=0.062)). Source data are provided as a Source Data file.
C. Schematic representation of changes in the expression of the *Dlk1-Dio3* cluster in $F1^{mat\text{-}HFD}$ as determined by QRT-PCR analysis, where blue arrows indicate fold-increase in transcript levels in the liver of P56 mice, as compared to $F1^{mat\text{-}CD}$ controls (based on data presented in Figure 4B). Expression of all genes increased in $F1^{mat\text{-}HFD}$ liver, with large increases in maternally-derived transcripts (such as *Gtl2, Rtl1as, Rian, Mirg*) that are normally expressed only minimally. Total *Dlk1* expression was found to be increased in $F1^{mat\text{-}HFD}$. (N=4 animals per group). Source data are provided as a Source Data file.

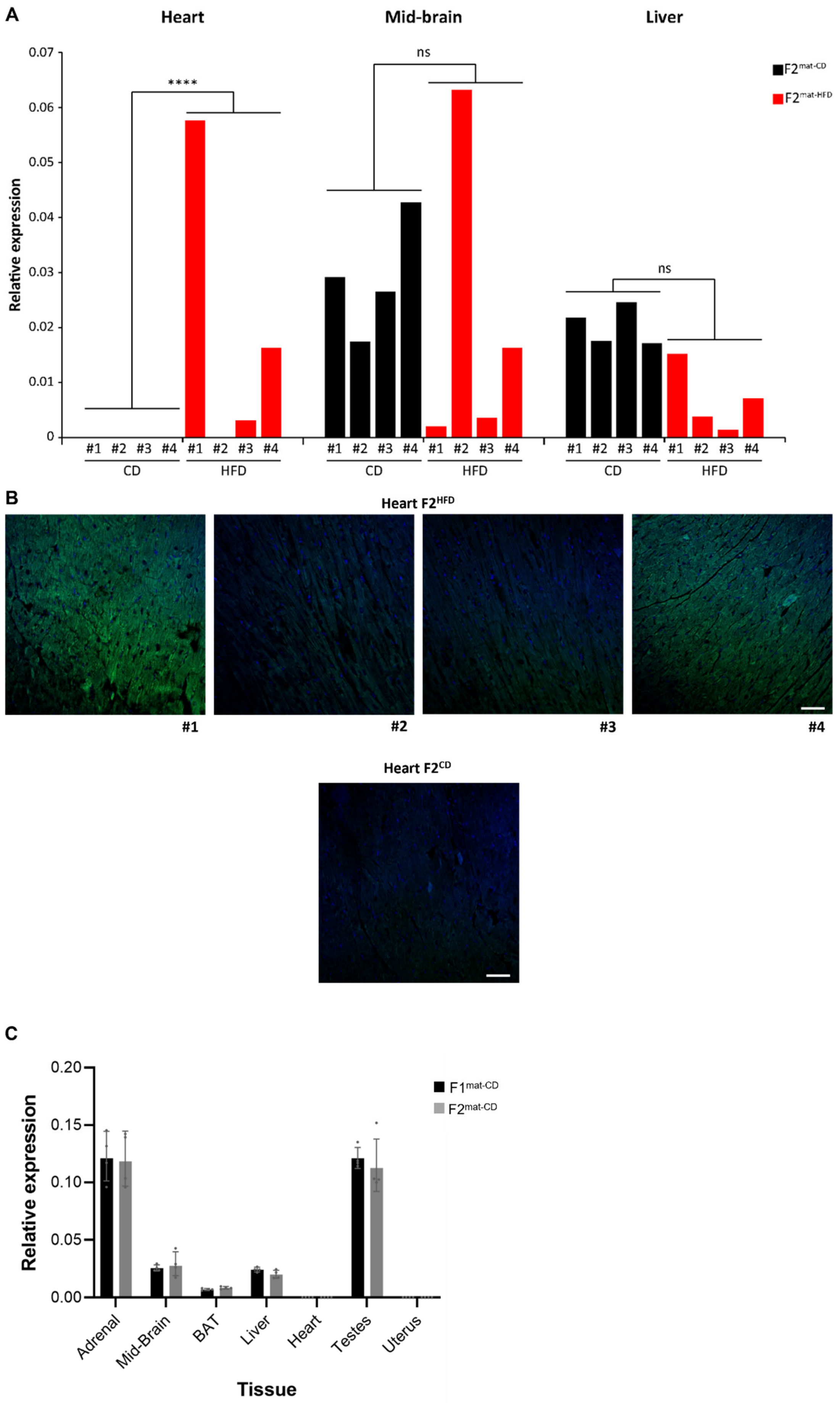
A
Heart
Mid-brain
Liver
Relative expression
0.07
0.06
0.05
0.04
0.03
0.02
0.01
0
****
ns
ns
F2mat-CD
F2mat-HFD
#1 #2 #3 #4 #1 #2 #3 #4
CD
HFD
B
Heart F2HFD
#1
#2
#3
#4
Heart F2CD
C
Relative expression
0.20
0.15
0.10
0.05
0.00
F1mat-CD
F2mat-CD
Adrenal
Mid-Brain
BAT
Liver
Heart
Testes
Uterus
Tissue

Figure S4. Heterogenous and ectopic expression of *Dlk1* in F2$^{mat\text{-}HFD}$ animals

A. *Dlk1* expression determined by QRT-PCR in organs from individual F2$^{mat\text{-}CD}$ (black) and F2$^{mat\text{-}HFD}$ (red) animals, corresponding to Figure 5D. Expression levels were normalised to *β-Actin*, *18S* and *Hprt* expression. Variable levels of *Dlk1* were observed within tissues between samples of F2$^{mat\text{-}HFD}$ animals. (Statistics correspond to the results shown in Figure 5D from Holm-Šídák's multiple comparisons test for the effect of diet in each tissue: ****padj<0.0001, ns=not significant). (Geometric SD Mid-brain: CD 1.444, HFD 4.718; Liver: CD 1.191, HFD 2.757; Heart CD 2.062, HFD 16.142). Source data are provided as a Source Data file.
B. Immunolabelling of Dlk1 (green) and DAPI stain (blue) in F2$^{mat\text{-}HFD}$ (upper panels) and F2$^{mat\text{-}CD}$ (lower panel) cardiac tissue. Samples correspond to those used in Figure S4A and confirm variable Dlk1 expression between individual F2$^{mat\text{-}HFD}$ mice. Images were taken with identical gain and exposure times between samples. Scale bars represent 50 µm. Sections were generated from four F2$^{mat\text{-}HFD}$ and two F2$^{mat\text{-}CD}$ animals. Staining was performed twice for each individual; the F2$^{mat\text{-}CD}$ image is representative.
C. QRT-PCR data from Figures 4C (F1 generation) and 5D (F2 generation) were analysed together to compare *Dlk1* expression between generations. The graph shows relative *Dlk1* expression levels (normalised to *β-Actin*, *18S* and *Hprt*) for F1$^{mat\text{-}CD}$ (black) and F2$^{mat\text{-}CD}$ (dark grey) P56 males (or females for uterus samples). (Bars show the geometric mean of relative expression with geometric SD; N=4+4 individual mice). Generation (F1 vs F2) was not a significant source of variation in *Dlk1* expression (Two-way ANOVA on delta-Ct values (Tissue p<0.0001, Generation p=0.1132, Interaction p=0.0337)). Source data are provided as a Source Data file.

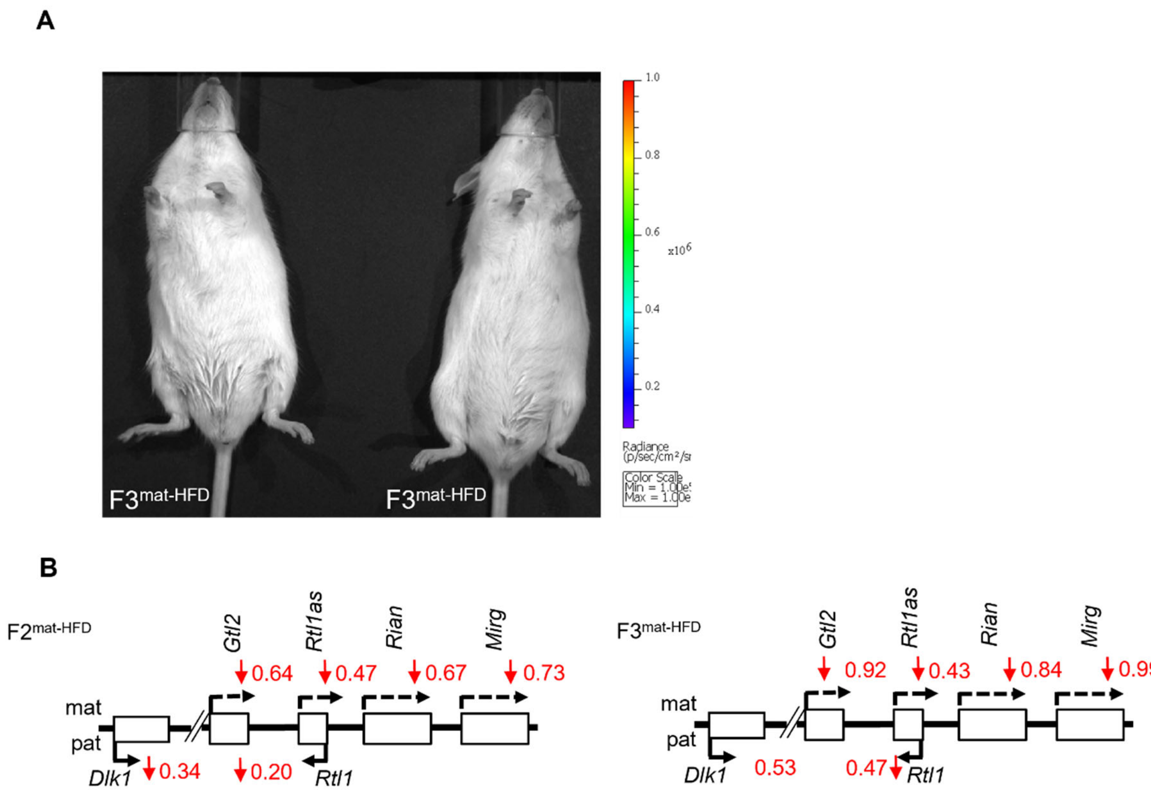

Figure S5. Imprinted *Dlk1* expression is largely restored in the F3 generation

A. BL imaging of two representative *Dlk1-FLucLacZ* F3$^{mat\text{-}HFD}$ P56 male mice, where minimal luciferase activity was detected (levels comparable with KI$^{mat\text{-}CD}$ animals).
B. Schematic representation of fold changes in gene expression, as analysed by QRT-PCR, at the *Dlk1-Dio3* cluster in F2$^{mat\text{-}HFD}$ (left) and F3$^{mat\text{-}HFD}$ (right) liver when compared to F2$^{mat\text{-}CD}$, showing a generalised reduction in transcript levels (red arrows). Expression was normalised to *β-Actin* for this single tissue comparison. (N=4 animals per group). Source data are provided as a Source Data file.

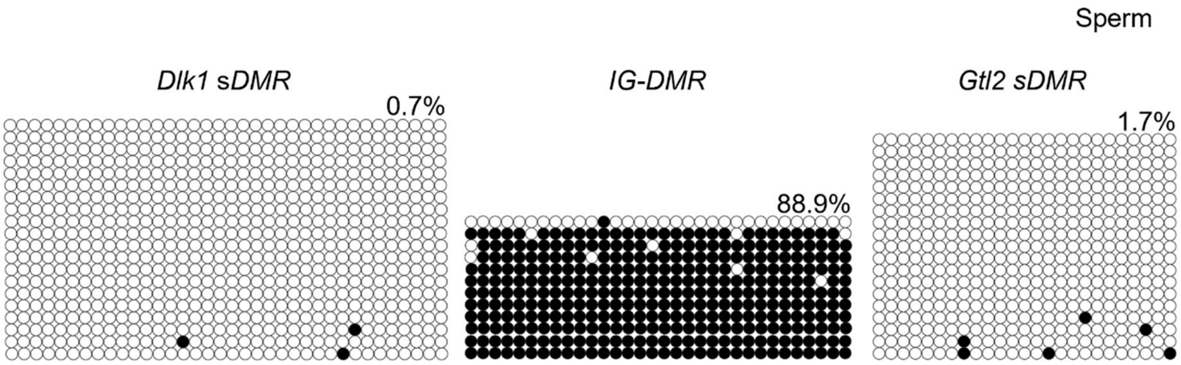

Figure S6. Methylation of *Dlk1-Dio3* cluster DMRs is unchanged in F1$^{mat\text{-}HFD}$ sperm

Bisulphite analysis showing DNA methylation at *Dlk1 sDMR* (left), *IG-DMR* (middle) and *Gtl2 sDMR* (right) in F1$^{mat\text{-}HFD}$ P56 sperm from representative individuals. The *IG-DMR* was found to be hyper-methylated, while both the *Dlk1 sDMR* and *Gtl2 sDMR* were found to be largely un-methylated. Closed circles indicate methylated CpGs and open circles un-methylated CpGs, with each row representing an individual clone. Percentages indicate total methylation of the analysed region from two animals. Source data are provided as a Source Data file.

A

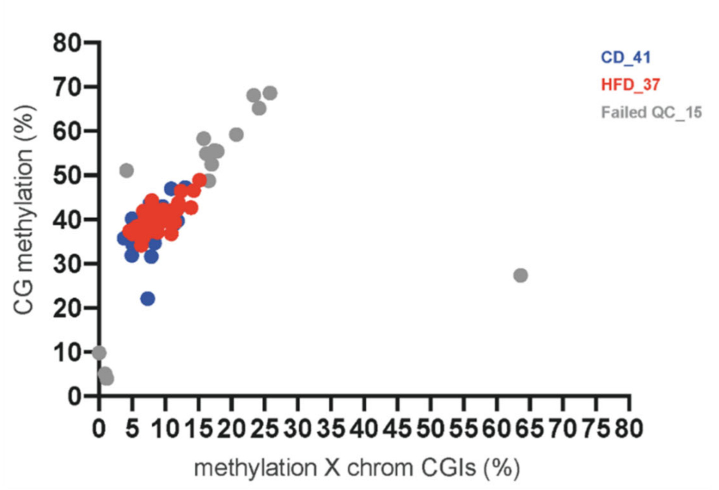

B

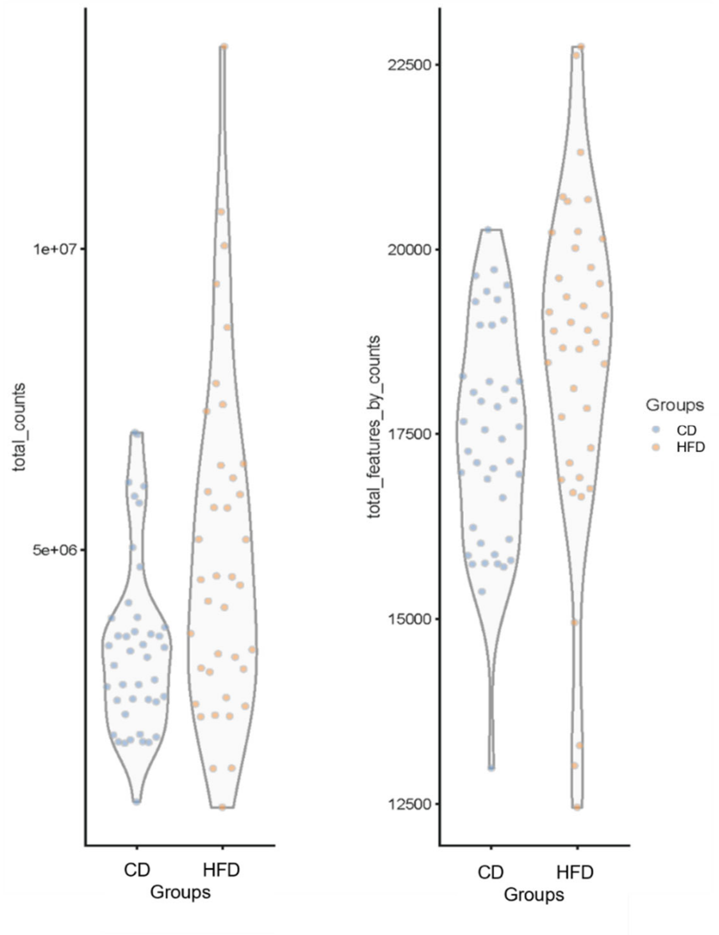

Figure S7. scAnalysis quality control

A. Methylation level of X chromosome observed in individual oocytes between F1$^{mat\text{-}CD}$ and F1$^{mat\text{-}HFD}$, with the number of oocytes that passed quality control listed (41 + 37), in addition to those that failed (15). Thresholds were set at 16% for CGI methylation on the X chromosome and 50% for global CpG methylation.
B. Total counts observed in individual oocytes from F1$^{mat\text{-}CD}$ and F1$^{mat\text{-}HFD}$ F1 females for RNA-seq analysis.

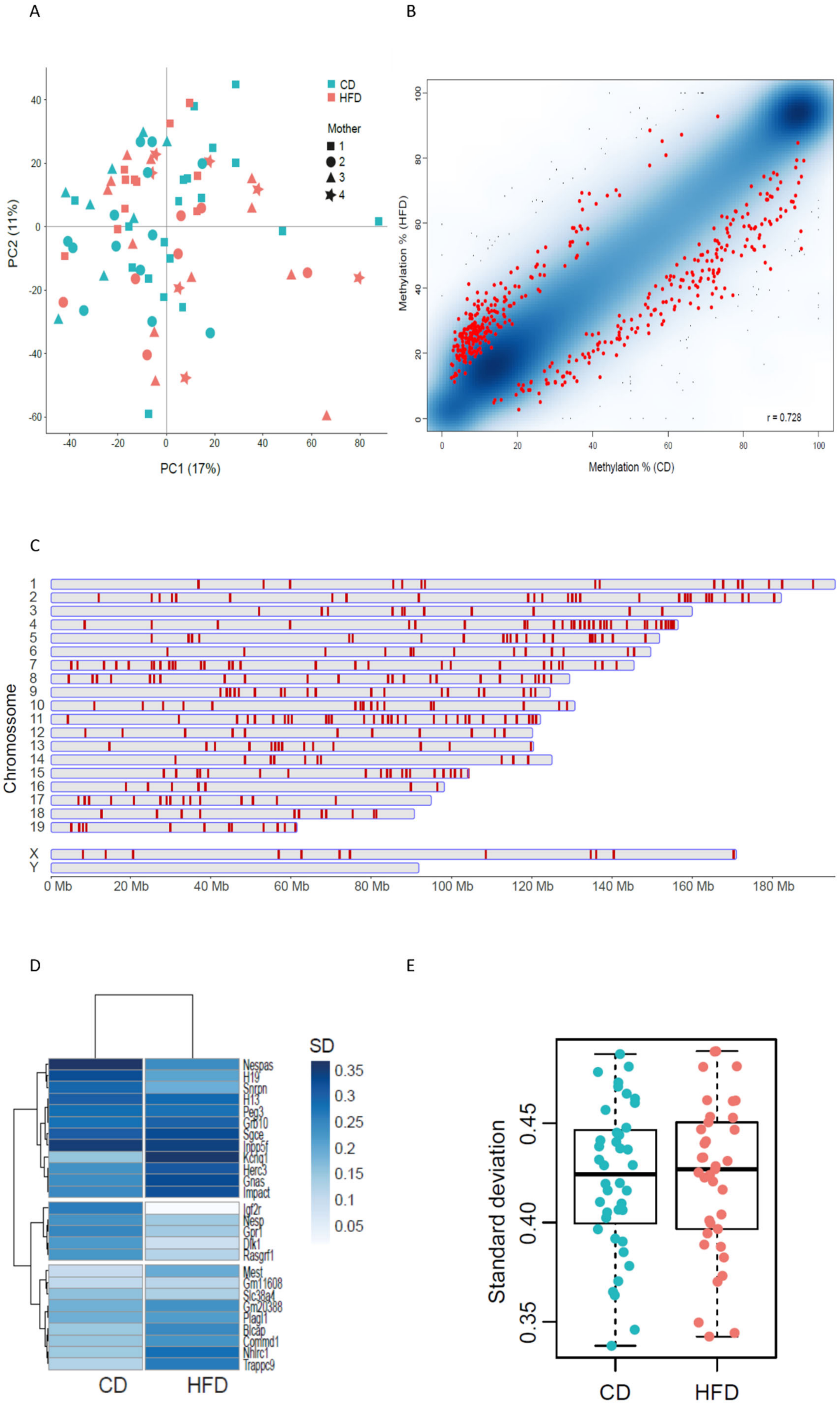
A
CD
HFD
Mother
1
2
3
4
PC2 (11%)
PC1 (17%)
B
Methylation % (HFD)
Methylation % (CD)
r = 0.728
C
Chromossome
0 Mb
20 Mb
40 Mb
60 Mb
80 Mb
100 Mb
120 Mb
140 Mb
160 Mb
180 Mb
D
SD
Nespas
H19
Snrpn
H13
Peg3
Grb10
Sgce
Inpp5f
Kcnq1
Herc3
Gnas
Impact
Igf2r
Nesp
Gpr1
Dlk1
Rasgrf1
Mest
Gm11608
Slc38a4
Gm20388
Plagl1
Blcap
Commd1
Nhlrc1
Trappc9
CD
HFD
E
Standard deviation
CD
HFD

Figure S8. scDNA-methylation analysis of F1$^{mat\text{-}HFD}$ oocytes

A. PCA plot of the 41 and 37 scBS-seq datasets of oocytes from F1$^{mat\text{-}CD}$ and F1$^{mat\text{-}HFD}$ females, respectively. The plot is based on 100-CpG running windows with 2 kb spacing containing reads in all scBS-seq datasets.
B. Scatterplot of grouped data demarking the 439 100-CpG tiles (red) called differentially methylated between F1$^{mat\text{-}CD}$ and F1$^{mat\text{-}HFD}$ with an absolute methylation difference of ≥10%. The 439 DMRs represent DMRs identified as significant at an FDR of <0.05, with at least 10% difference in methylation between groups, in 70% of 100 permutations of 36 cells in pools of 9 cells per group.
C. Chromosome view showing distribution of DMRs.
D. Heatmap representing the variation (SD) in methylation of each gDMR across all the oocytes in the F1$^{mat\text{-}CD}$ and F1$^{mat\text{-}HFD}$ groups.
E. Box-whisker plots representing the variation (SD) of all the informative gDMRs in each individual oocyte in the CD (N=41) and HFD (N=37) groups. Each point represents a single oocyte, whiskers represent the minima and maxima values, boxes represent the interquartile ranges, and horizontal lines represent the medians.

A

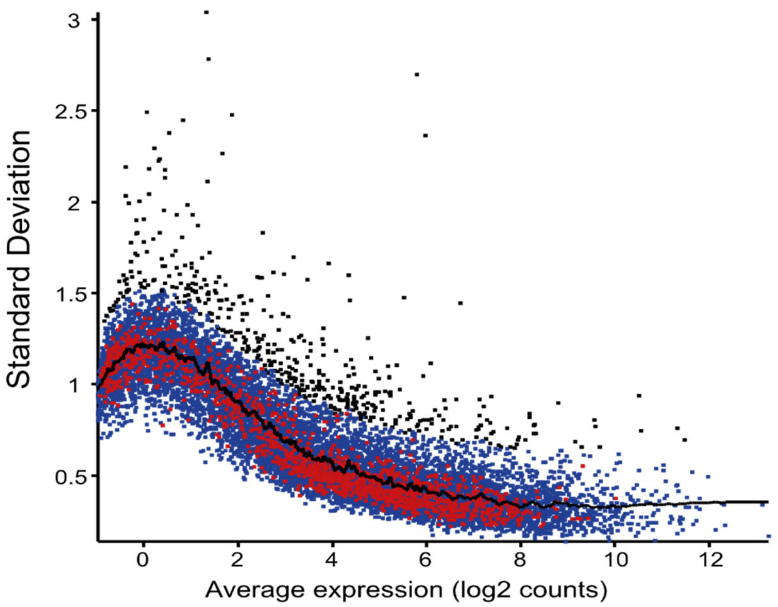

B

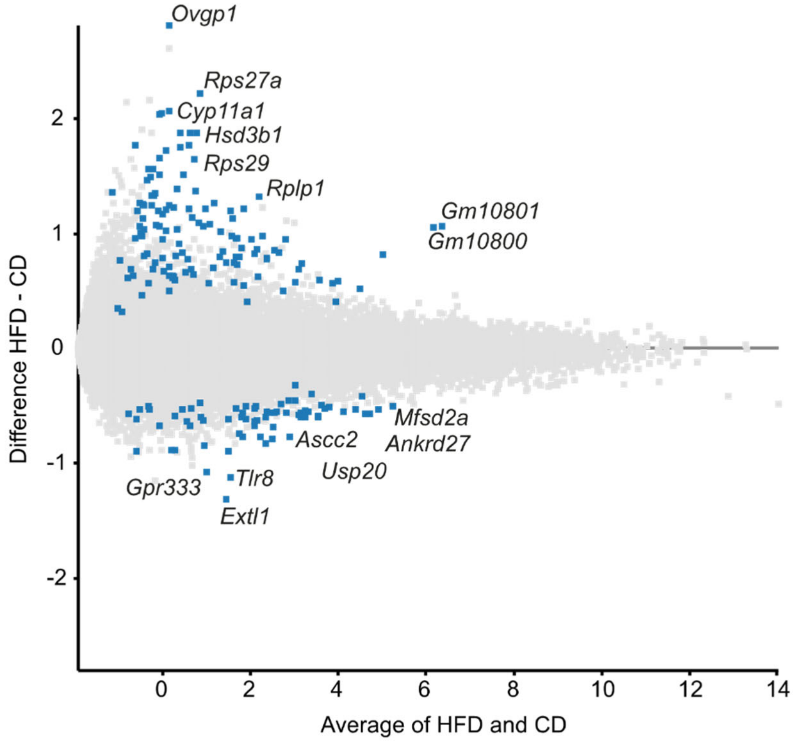

Figure S9. scRNA-seq analysis of F1$^{mat\text{-}HFD}$ oocytes

A. Variance plot highlighting in black the 166 most variable genes (>0.528-fold from the mean standard deviation) in all samples.
B. MA plot showing the difference in expression between F1$^{mat\text{-}CD}$ and F1$^{mat\text{-}HFD}$ oocytes of the 166 most variable genes (highlighted in blue).

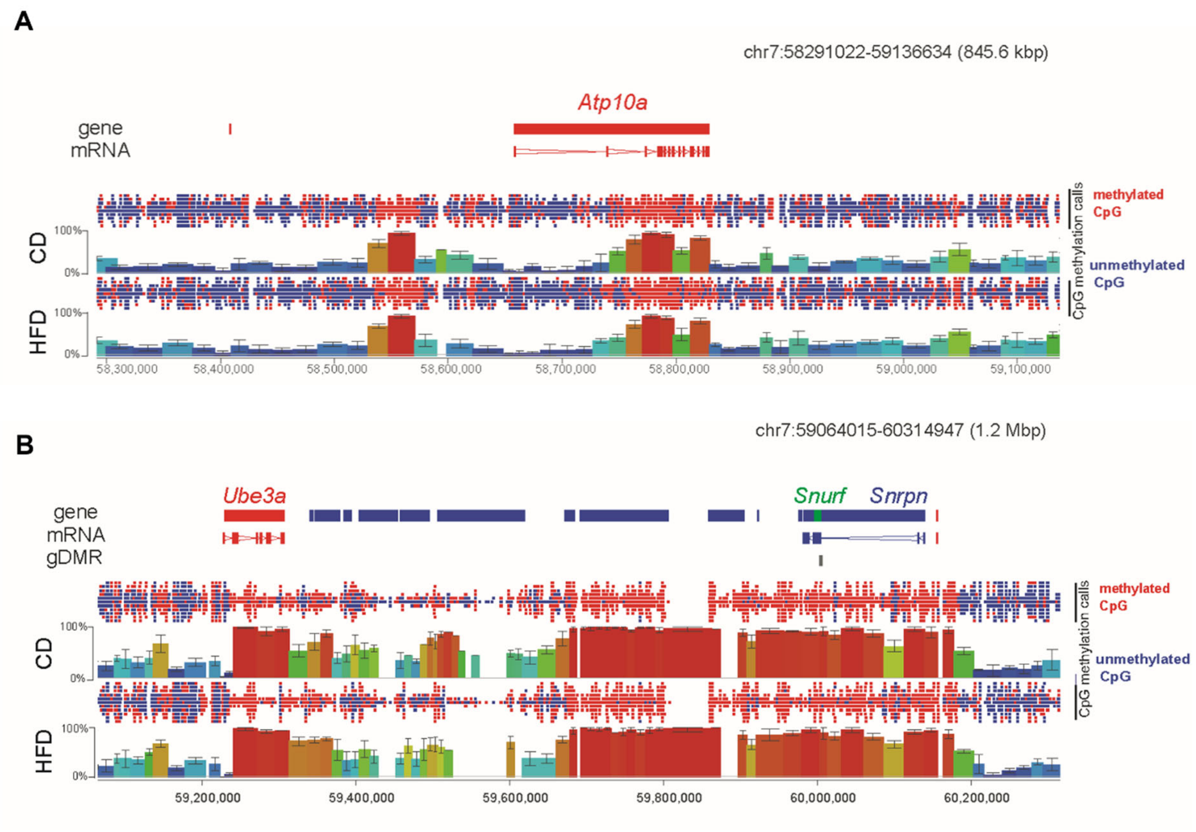

Figure S10. scDNA-methylation analysis of F1$^{mat\text{-}HFD}$ oocytes

A. SeqMonk screenshot showing mean DNA methylation in F1$^{mat\text{-}CD}$ and F1$^{mat\text{-}HFD}$ oocytes over non-overlapping 100 CpG windows (colour-coded blocks) and individual CpG methylation calls (methylated red; un-methylated blue) at the *Atp10a* non-imprinted locus. Error bars represent SD from the mean of 5 pseudo-bulk groupings of 7-8 cells each.
B. SeqMonk screenshot showing mean DNA methylation in F1$^{mat\text{-}CD}$ and F1$^{mat\text{-}HFD}$ oocytes across the *Ube3a*-*Snrpn* imprinted cluster (displayed as in panel A).
