# Supplementary material for: Epigenetic changes induced by in utero dietary challenge result in phenotypic variability in successive generations of mice
Source: Nat Commun. 2022 May 5;13:2464. doi: 10.1038/s41467-022-30022-2 (PMC9072353; doi:10.1038/s41467-022-30022-2)
Supplement: Supplementary file 2 — Description of additional Supplementary Files [file 41467_2022_30022_MOESM2_ESM.pdf]

Descriptions of additional Supplementary Data Files

Supplementary Movies 1-3. Optical Projection Tomography of Dlk1 expression in E11.5 embryos Optical Projection Tomography (OPT) of LacZ stained E11.5 Dlk1-FLucLacZ embryos. Absorbance (green) was measured in the liver, cartilage, gonadal ridges, and a subset of forebrain regions in KIpat (Movie 2) embryos. Absorbance was weaker in KImat (Movie 3) embryos, with very low signal level detected in wt (Movie 1) embryos.

Supplementary Data 1. scRNA-Seq analysis of F1mat-HFD oocytes Excel file containing the 166 most variably expressed genes and those found to be up- or down-regulated in F1mat-HFD compared to F1mat-CD oocytes.
