# Supplementary figures and images for: Epigenetic changes induced by in utero dietary challenge result in phenotypic variability in successive generations of mice
Source: Nat Commun. 2022 May 5;13:2464. doi: 10.1038/s41467-022-30022-2 (PMC9072353; doi:10.1038/s41467-022-30022-2)

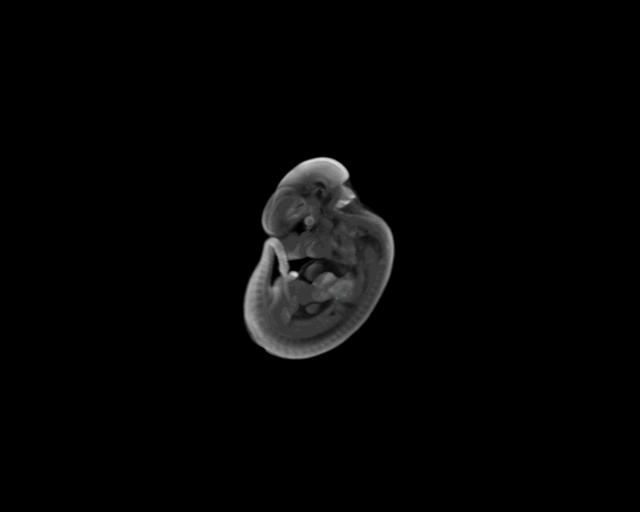

Supplement: Supplementary file 3 — Supplementary Movie 1 [file 41467_2022_30022_MOESM3_ESM.gif]

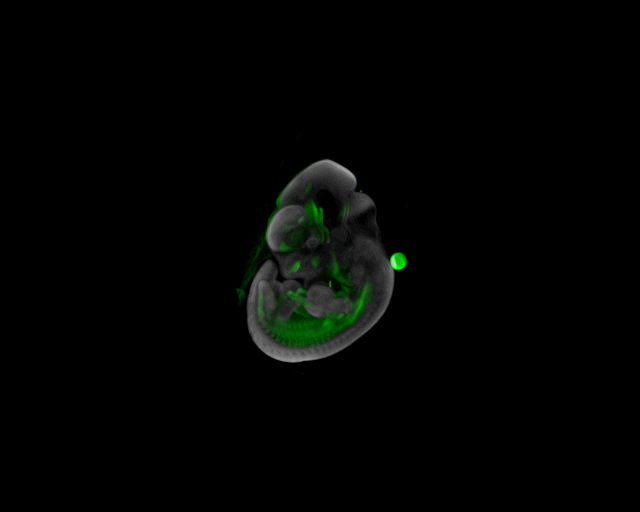

Supplement: Supplementary file 4 — Supplementary Movie 2 [file 41467_2022_30022_MOESM4_ESM.gif]

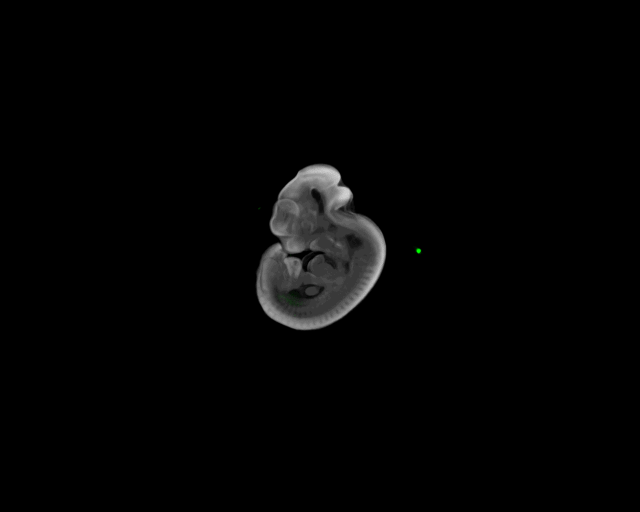

Supplement: Supplementary file 5 — Supplementary Movie 3 [file 41467_2022_30022_MOESM5_ESM.gif]
