# Supplementary material for: Epigenetic changes induced by in utero dietary challenge result in phenotypic variability in successive generations of mice
Source: Nat Commun. 2022 May 5;13:2464. doi: 10.1038/s41467-022-30022-2 (PMC9072353; doi:10.1038/s41467-022-30022-2)
Supplement: Supplementary file 7 — Reporting Summary [file 41467_2022_30022_MOESM7_ESM.pdf]

nature portfolio

Corresponding author(s): Amanda G Fisher

Last updated by author(s): Apr 4, 2022

Reporting Summary

Nature Portfolio wishes to improve the reproducibility of the work that we publish. This form provides structure for consistency and transparency in reporting. For further information on Nature Portfolio policies, see our Editorial Policies and the Editorial Policy Checklist.

Statistics

For all statistical analyses, confirm that the following items are present in the figure legend, table legend, main text, or Methods section.

n/a	Confirmed	
☐	☒	The exact sample size (*n*) for each experimental group/condition, given as a discrete number and unit of measurement
☐	☒	A statement on whether measurements were taken from distinct samples or whether the same sample was measured repeatedly
☐	☒	The statistical test(s) used AND whether they are one- or two-sided<br>*Only common tests should be described solely by name; describe more complex techniques in the Methods section.*
☐	☒	A description of all covariates tested
☐	☒	A description of any assumptions or corrections, such as tests of normality and adjustment for multiple comparisons
☐	☒	A full description of the statistical parameters including central tendency (e.g. means) or other basic estimates (e.g. regression coefficient) AND variation (e.g. standard deviation) or associated estimates of uncertainty (e.g. confidence intervals)
☐	☒	For null hypothesis testing, the test statistic (e.g. *F*, *t*, *r*) with confidence intervals, effect sizes, degrees of freedom and *P* value noted<br>*Give P values as exact values whenever suitable.*
☒	☐	For Bayesian analysis, information on the choice of priors and Markov chain Monte Carlo settings
☒	☐	For hierarchical and complex designs, identification of the appropriate level for tests and full reporting of outcomes
☒	☐	Estimates of effect sizes (e.g. Cohen's *d*, Pearson's *r*), indicating how they were calculated

Our web collection on statistics for biologists contains articles on many of the points above.

Software and code

Policy information about availability of computer code

Data collection	Bioluminescence images were acquired with Living Image software 4.5 (Caliper Life Sciences).<br>RT-PCR data were acquired with a Bio-Rad CFX96 system.<br>Immunofluorescence images were acquired with a LSM880 confocal microscope (Zeiss).<br>Slides were imaged with NanoZoomer-XR (Hamamatsu).<br>OPT images were acquired with a CCD camera (Clara, Andor Technology Ltd) using a telecentric zoom lens (modules NT56-625, NT59-671 and NT59-672, Edmund Optics Ltd). Transmitted light images were acquired through a 716±20 nm band-pass filter (FF01-716/40-25, Laser 2000 UK Ltd). Fluorescence OPT imaging was performed using a 473 nm excitation source (Cobolt BluesTM, Cobolt AB), imaging at 520±17 nm (FF01520/35-25, Laser 2000 UK Ltd).<br>Pyrosequencing was performed with the PyroMark Q96 MD pyrosequencer (Qiagen).<br>Small RNA library sequencing was performed on a MiSeq (Illumina).<br>Single cell library sequencing was performed on a NextSeq 5000 (Illumina).

Data analysis	Bioluminescence images were analysed with Living Image software 4.5 (Caliper Life Sciences).<br>Immunofluorescence images analysed with Zen Blue 3.4 (Zeiss).<br>Immunohistochemistry images were analysed with NDP.view2 (Hamamatsu).<br>For scBS-seq analysis, read alignment, deduplication and methylation calling was performed using Bismark v0.22.1.<br>For scRNA-seq analysis, reads were mapped with hisat2 v2.1.0. Gene expression was analysed with SeqMonk v1.46.0 [https://www.bioinformatics.babraham.ac.uk/projects/seqmonk/]. Principal component analysis and hierarchical clustering was performed using R (R Core Team, 2016). GO analysis was done using Gene Ontology enRIchment analysis and visualizAtion tool (GOrilla) [http://cbl-gorilla.cs.technion.ac.il/l], followed by reduction of terms with Revigo [http://revigo.irb.hr].<br>Small RNA-seq libraries were analysed using sRNAtoolbox [https://arn.ugr.es/srnatoolbox/srnabench/].<br>Microsoft Excel and GraphPad Prism (v9.2.0) were used for calculations, statistical analysis and the preparation of graphs.

For manuscripts utilizing custom algorithms or software that are central to the research but not yet described in published literature, software must be made available to editors and reviewers. We strongly encourage code deposition in a community repository (e.g. GitHub). See the Nature Portfolio guidelines for submitting code & software for further information.

Data

Policy information about availability of data

All manuscripts must include a data availability statement. This statement should provide the following information, where applicable:

- Accession codes, unique identifiers, or web links for publicly available datasets
- A description of any restrictions on data availability
- For clinical datasets or third party data, please ensure that the statement adheres to our policy

Single cell sequencing data generated in this study have been deposited in the GEO database under accession code GSE175538 [https://www.ncbi.nlm.nih.gov/geo/query/acc.cgi?acc=GSE175538]. Raw data for all graphs and tables generated in this study are provided in the Source Data file. Other original data can be requested from the corresponding author upon reasonable grounds.

Field-specific reporting

Please select the one below that is the best fit for your research. If you are not sure, read the appropriate sections before making your selection.

☒ Life sciences ☐ Behavioural & social sciences ☐ Ecological, evolutionary & environmental sciences

For a reference copy of the document with all sections, see nature.com/documents/nr-reporting-summary-flat.pdf

Life sciences study design

All studies must disclose on these points even when the disclosure is negative.

Sample size	Sample sizes were set to ensure reproducible results. For reporter line validation, at least 3 independent litters were generated for each embryonic and adult time-point, which was sufficient to ensure observations were reproducible. For generational studies and production of F1 and F2 animals, a minimum of 6 female mice were set-up from the F0 and F1 generations, allowing the phenotype to be observed, while also ensuring that excess animals were not used (NC3Rs). For observations of the F3 generation, 3 female mice and 2 male mice were separately set up, providing sufficient F3 offspring to determine that no bioluminescent phenotype could be identified. For QRT-PCR analysis, RNA was extracted from tissues from 4 individuals, providing sufficient numbers for statistical comparisons between groups. Staining was performed on a minimum of 2 individuals, ensuring observations were reproducible; and at least 4 F2-matHFD individuals to observe variability. Bisulphite analysis was performed on two individuals, providing sufficient clones for statistical comparison and calculation of percentage methylation, and ensuring that observations were reproducible between individuals. For the single cell analysis, oocytes from 6 animals per group were taken, producing sufficient oocytes to produce allow robust statistical analysis. For the small RNA analysis, oocytes from 4 animals per group were taken, allowing robust statistical analysis between groups.
Data exclusions	15 individual oocyte data sets were excluded, as these failed QC, determined by inappropriate global and/or X-chromosome methylation levels. No other data were excluded.
Replication	For reporter line validation, at least 3 independent litters were generated for each embryonic and adult time-point. For generational studies and production of F1 and F2 animals, a minimum of 6 female mice were set-up from the F0 and F1 generations. For observations of the F3 generation, 3 female mice and 2 male mice were separately set up. Staining experiments were performed on a minimum of 2 individuals, and staining was performed in duplicate for each individual. For QRT-PCR each well was pipetted in technical triplicate and each plate run in technical duplicate, with four samples per group. Bisulphite analysis was repeated twice. For the single cell analysis, oocytes from 6 animals per group were taken, producing a total of 41 F1mat-CD and 37 F1mat-HFD informative datasets. For the small RNA analysis, libraries were generated from oocytes taken from 4 animals per group.<br>All replicate attempts were successful, apart from the exclusion of 15 single oocyte datasets as indicated above, and one F1mat-HFD female set up with a wtCD male did not produce any F2 litters (reported in the manuscript).
Randomization	Female mice were randomly assigned to experimental (dietary) group. No other experiments involved allocation to experimental groups.
Blinding	Researchers were blinded to groups during data acquisition, including in vivo imaging and molecular biology. Researchers were not blinded during analysis since the data were analysed according to defined comparisons.

Reporting for specific materials, systems and methods

We require information from authors about some types of materials, experimental systems and methods used in many studies. Here, indicate whether each material, system or method listed is relevant to your study. If you are not sure if a list item applies to your research, read the appropriate section before selecting a response.

Materials & experimental systems

n/a	Involved in the study	
☐	☒	Antibodies
☒	☐	Eukaryotic cell lines
☒	☐	Palaeontology and archaeology
☐	☒	Animals and other organisms
☒	☐	Human research participants
☒	☐	Clinical data
☒	☐	Dual use research of concern

Methods

n/a	Involved in the study	
☒	☐	ChIP-seq
☒	☐	Flow cytometry
☒	☐	MRI-based neuroimaging

Antibodies

Antibodies used	Anti-Dlk1 [3A10]: ab119930 (Abcam) Mouse monoclonal.<br>Recombinant Anti-Firefly Luciferase [EPR17790]: ab185924 (Abcam) Rabbit monoclonal.<br>Goat anti-rabbit 568: A-11011 (Thermo Scientific).<br>Goat anti-mouse 488: A-10680 (Thermo Scientific).<br>Goat anti-mouse HRP-conjugated. #31430 (Thermo Scientific).
Validation	Anti-Dlk1 is covered by the Abpromise guarantee for use in ICC/IF and is determined to react with mouse Dlk1 (supplier website).<br>Anti-Firefly Luciferase is covered by the Abpromise guarantee for use in ICC/IF and is determined to react with Firefly Luciferase and does not react with mouse (supplier website).<br>Anti-Dlk1 and Anti-firefly Luciferase antibodies were tested on positive and negative control samples, and with secondary-only controls.

Animals and other organisms

Policy information about studies involving animals; ARRIVE guidelines recommended for reporting animal research

Laboratory animals	Mus Musculus, B6(Cg)-Tyrc-2J/J, males and females, embryonic time-points (E11.5, E14.5, E17.5) and adults (4, 6, 8, 10 weeks). Mice were housed on a 12-hour light-dark cycle with a temperature range of 21 +/- 2 C and humidity range of 55 +/- 10 % in pathogen free conditions.
Wild animals	No wild animals were used in this study.
Field-collected samples	No field-collected samples were used in this study.
Ethics oversight	Imperial College AWERB committee. Work was performed under a UK Home Office Project Licence.

Note that full information on the approval of the study protocol must also be provided in the manuscript.
